# Tissue Equivalent Curved Organic X‐ray Detectors Utilizing High Atomic Number Polythiophene Analogues

**DOI:** 10.1002/advs.202304261

**Published:** 2023-11-02

**Authors:** M. Prabodhi A. Nanayakkara, Qiao He, Arvydas Ruseckas, Anushanth Karalasingam, Lidija Matjacic, Mateus G. Masteghin, Laura Basiricò, Ilaria Fratelli, Andrea Ciavatti, Rachel C. Kilbride, Sandra Jenatsch, Andrew J. Parnell, Beatrice Fraboni, Andrew Nisbet, Martin Heeney, K. D. G. Imalka Jayawardena, S. Ravi P. Silva

**Affiliations:** ^1^ Advanced Technology Institute, Department of Electrical and Electronic Engineering University of Surrey Guildford Surrey GU2 7XH UK; ^2^ Department of Chemistry and Centre for Processable Electronics Imperial College London, White City Campus London W12 0BZ UK; ^3^ School of Physics & Astronomy University of St Andrews Physical Science Building, North Haugh St Andrews UK; ^4^ Sri Lanka Institute of Nanotechnology Pitipana – Thalagala Rd Homagama Sri Lanka; ^5^ National Physical Laboratory Teddington Middlesex TW11 0LW UK; ^6^ Department of Physics and Astronomy University of Bologna Viale Berti Pichat 6/2 Bologna 40127 Italy; ^7^ National Institute for Nuclear Physics INFN Section of Bologna Bologna Italy; ^8^ Department of Chemistry University of Sheffield Dainton Building Sheffield S3 7HF UK; ^9^ FLUXiM AG Katharina‐Sulzer‐Platz 2 Winterthur 8400 Switzerland; ^10^ Department of Physics and Astronomy University of Sheffield Hicks Building Sheffield S3 7RH UK; ^11^ Department of Medical Physics and Biomedical Engineering University College London Gower St, Bloomsbury London WC1E 6BT UK

**Keywords:** flexible, heteroatom, organic electronics, photonics, x‐ray detectors

## Abstract

Organic semiconductors are a promising material candidate for X‐ray detection. However, the low atomic number (Z) of organic semiconductors leads to poor X‐ray absorption thus restricting their performance. Herein, the authors propose a new strategy for achieving high‐sensitivity performance for X‐ray detectors based on organic semiconductors modified with high –Z heteroatoms. X‐ray detectors are fabricated with p‐type organic semiconductors containing selenium heteroatoms (poly(3‐hexyl)selenophene (P3HSe)) in blends with an n‐type fullerene derivative ([6,6]‐Phenyl C71 butyric acid methyl ester (PC_70_BM). When characterized under 70, 100, 150, and 220 kVp X‐ray radiation, these heteroatom‐containing detectors displayed a superior performance in terms of sensitivity up to 600 ± 11 nC Gy^−1^ cm^−2^ with respect to the bismuth oxide (Bi_2_O_3_) nanoparticle (NP) sensitized organic detectors. Despite the lower Z of selenium compared to the NPs typically used, the authors identify a more efficient generation of electron‐hole pairs, better charge transfer, and charge transport characteristics in heteroatom‐incorporated detectors that result in this breakthrough detector performance. The authors also demonstrate flexible X‐ray detectors that can be curved to a radius as low as 2 mm with low deviation in X‐ray response under 100 repeated bending cycles while maintaining an industry‐standard ultra‐low dark current of 0.03 ± 0.01 pA mm^−2^.

## Graphical abstract

Organic semiconductors are a promising class of materials for X‐ray detection. However, their performance has been restricted by poor X‐ray attenuation. This work introduces a methodology to achieve organic X‐ray detectors with high sensitivity via high Z heteroatom inclusion that leads to additional benefit of tissue equivalence and conformable functionality.

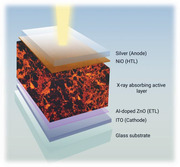

## 1 Introduction

Over the last two decades, there have been a steady increase in the use of X‐ray radiation detectors in a wide span of applications such as medical imaging (radiography, mammography, and computed tomography), clinical radiotherapy dosimetry, industrial inspection, security, personal radiation protection, and cultural heritage preservation. Most of these applications consist of “objects” of complicated shape and geometries, which demands for X‐ray radiation detectors of flexible and large area configuration. However, none of the commercially available detectors are capable of catering to these requirements. Most of the commercially available direct X‐ray detectors consist of elemental semiconductors (silicon and germanium) and compound semiconductors (cadmium telluride, cadmium zinc telluride, and gallium arsenide) which are capable of delivering an exceptional performance, but are heavy, rigid and require high power supply with costly manufacturing procedures to realize large area detection. This accentuates the need for alternate detector strategies that can enable flexible, large area functionality under low cost, low power budget conditions.

Recently organic semiconductors have risen to prominence as a potential candidate for the fabrication of optoelectronic devices (e.g., solar cells,^[^ ^1^ ^]^ radiation detectors,^[^ ^2^ ^]^ and LEDs^[^ ^3^ ^]^) fueled by their possibility of realizing large area flexible devices by using environmentally friendly, low cost, low temperature solution deposition techniques.^[^ ^4^, ^5^, ^6^ ^]^ Particularly, organic semiconductors have demonstrated favorable characteristics as an X‐ray absorber for direct detection of radiation, regardless of their low X‐ray absorption and charge transport properties compared to the inorganic counterparts. In fact, there have been several reports on using thick films of conjugated polymers such as poly(triaryl amine) (PTAA),^[^ ^7^ ^]^ organic semiconducting single crystals such as 4‐hydroxycyanobenzene (4HCB) and 1,8‐naphthalene imide (NTI),^[^ ^8^, ^9^ ^]^ and blends of conjugated polymers and small organic molecules such as the blend of PTAA and 6,13‐bis(triisopropylsilylethinyl)pentacene (TIPS‐Pentacene)^[^ ^10^ ^]^ for the fabrication of state‐of‐the‐art direct X‐ray detectors.

However, utilizing organic semiconductors for X‐ray detection to their full extent has been challenging due to the low X‐ray attenuation coefficient of the organic material leading to poor sensitivities. Several strategies have been proposed and demonstrated, such as the incorporation of high atomic number (Z) direct converting nanoparticles (NPs),^[^ ^6^, ^11^, ^12^ ^]^ carbon nanotubes,^[^ ^13^ ^]^ and scintillating particles^[^ ^14^ ^]^ within the organic semiconductor matrix as an alternate approach toward enhancing the sensitivity. Among them, it is worth stating the hybrid X‐ray detector concept introduced by us^[^ ^6^, ^11^, ^12^ ^]^ where high – Z Bi_2_O_3_ NPs are incorporated into an organic bulk heterojunction (BHJ) which consist of the p‐type semiconductor Poly(3‐hexylthiophene‐2,5‐diyl) (P3HT) and n‐type [6,6]‐Phenyl C71 butyric acid methyl ester (PC_70_BM). Even though adopting the above mentioned strategies leads to an improvement in the detector performance, the inclusion of such X‐ray absorbing centers (i.e., high – Z NPs, carbon nanotubes, and scintillating particles) tends to degrade the electronic properties of the active layer.^[^ ^15^ ^]^ Moreover, high loading of such materials prevent the X‐ray absorbing active material from becoming tissue equivalent, which is a crucial requirement for medical dosimetry applications.^[^ ^9^, ^16^ ^]^ Therefore, it is important to examine new strategies to improve the X‐ray absorption cross section of the organic materials without sacrificing their performance.

In this work, we utilize a key benefit of organic semiconductors, i.e., the ability to tailor the organic semiconductor properties through heteroatom substitution to realize tissue‐equivalent organic X‐ray detectors. Based on the promising performance of P3HT, we investigated the heavier analogue in which the sulfur (S) heteroatom is replaced with selenium (Se), thus forming the p‐type polymer, poly(3‐hexyl)selenophene (P3HSe).^[^ ^17^, ^18^ ^]^ Compared to S, Se is larger and more polarizable, which can improve intermolecular contacts and charge carrier mobility. Se containing polymers also show a broader and more red‐shifted absorption spectrum compared to their thiophene analogue. Therefore, this approach not only enables the fabrication of a detector concept that is sensitive to X‐rays but also incorporates the advantages associated with low‐cost solution‐processable organic semiconductors including tissue equivalence. Particularly, the incorporation of high – Z elements such as selenium and tellurium into the backbone of conjugated polymers has prompted much interest with synthetic routes to such materials now being established.^[^ ^17^, ^19^, ^20^, ^21^ ^]^ P3HSe in particular has shown promising characteristics such as high device efficiencies and photocurrents for P3HSe: PC_70_BM BHJ solar cells^[^ ^18^, ^22^ ^]^ due to a crystalline morphology similar to its polythiophene analogue, which allows the desired nanoscale phase separation between p‐type P3HSe and n‐type PC_70_BM to be realized to ensure efficient charge separation and transport.^[^ ^17^, ^18^ ^]^

Herein, we demonstrate X‐ray detectors based on P3HSe: PC_70_BM blend films where the Se incorporation allows a broadband response and high sensitivity. This performance is astonishingly similar to that reported previously for our NP incorporated BHJ (NP‐BHJ) detectors^[^ ^6^, ^12^ ^]^ regardless of the lower Z of Se compared to Bi. Based on complementary characterization techniques, this is attributed to a number of benefits realized through heteroatom substitution including ultrafast and efficient charge separation and transfer, improved Schubweg values, and potentially more efficient secondary carrier generation as opposed to the use of high Z nanoparticles. Furthermore, the above advantages observed for rigid detectors are conveniently transferred to curved X‐ray detectors based on P3HSe: PC_70_BM that demonstrate less than 1.2% variation in X‐ray response even after 100 bending cycles when bent to a radius as small as 2 mm. Such features establish high – Z heteroatom modification as a promising strategy for achieving high performance X‐ray detectors based on organic semiconductors.

## 2 Results and Discussion

### 2.1 Energy Deposition, Charge Generation, and Charge Transfer Processes

**Figure 1:**
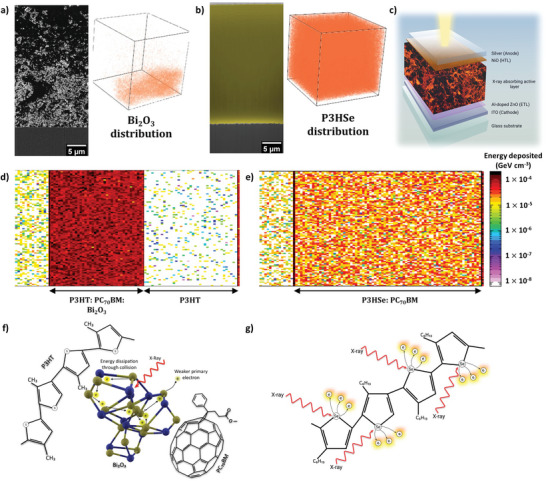
X‐ray energy deposition within the films. a) Cross‐sectional micrographs and the 3D renderings obtained from the ToF‐SIMS analysis showing the enrichment of Bi_2_O_3_ NPs at the bottom of the NP‐BHJ film, b) Cross‐sectional micrographs and the 3D renderings obtained from the ToF‐SIMS analysis indicating the uniform distribution of P3HSe across the P3HSe: PC_70_BM blend film, c) Schematic of the detector architecture used in this work. Energy deposited in the d) P3HT: PC_70_BM: Bi_2_O_3_ and e) P3HSe: PC_70_BM blend films simulated using the FLUKA software (http://www.fluka.org/fluka.php), f) Schematic diagram illustrating the photoelectric absorption in Bi_2_O_3_ complex which explains the electron energy dissipation process resulting in a weaker primary electron, g) Schematic diagram illustrating the photoelectric absorption of X‐ray photons by Se atoms in the P3HSe polymeric chain and generation of energetic primary electrons.

Prior to the identification of a suitable detector architecture, we evaluated the elemental distribution of Se within the P3HSe: PC_70_BM blend film and the distribution of Bi within the NP‐BHJ film. For this purpose, a combination of dual‐beam microscopy (FIB‐SEM) imaging of the absorber cross sections and time‐of‐flight secondary ion mass spectrometry (ToF‐SIMS) depth profiling was used. Similar to our previous work,^[^ ^6^, ^12^ ^]^ we observed the enrichment of Bi_2_O_3_ NPs toward the bottom half of the 55 µm thick NP‐BHJ blend (**Figure** **1**a). This is further supported by the ToF‐SIMS analysis carried out on the NP‐BHJ film (Figure 1a). This enrichment of Bi_2_O_3_ NPs at the bottom of the film can be explained by the sedimentation of NPs under the influence of gravitational forces in the absence of stabilizing functional groups. Contrary to this, P3HSe is observed to be uniformly distributed throughout the 55 µm thick film as supported by both backscattered electron cross‐sectional imaging as well as ToF‐SIMS analysis (Figure 1b). This is associated with the formation of a bulk heterojunction where the p‐type P3HSe and the n‐type PC_70_BM form a 3D interpenetrating bi‐continuous percolating network.

Following the above observations, we proceeded to evaluate the characteristics of these detectors based on the inverted device architecture. The use of an inverted device architecture is based on our previous work^[^ ^6^, ^12^ ^]^ where we show that the enrichment of the p‐type P3HT at the NP‐BHJ film surface acts as a charge blocking layer, thereby reducing the dark current to industrially relevant values.^[^ ^23^ ^]^ Adoption of device architectures based on organic blocking layers has been used for other hybrid and perovskite detector concepts to enable a low dark current response.^[^ ^24^ ^]^ Herein, the device stack (Figure 1c) used consists of glass/ indium tin oxide (ITO)/ Aluminium doped zinc oxide (Al‐doped ZnO)/ X‐ray absorbing active layer/ nickel oxide (NiO) / silver (Ag). The use of NiO as a hole transport layer (HTL) is motivated by the observation of the homogenous distribution of P3HSe throughout the detector volume (as discussed above). Hence the use of an HTL minimizes the charge recombination at the anode. The NP‐BHJ X‐ray detectors were also fabricated based on the above given layer stacking. To verify that any performance enhancement effects are solely generated by the incorporation of Se within the polymer backbone, X‐ray detectors based on a P3HT: PC_70_BM blend were also fabricated as a reference. All the X‐ray absorber active layer films were processed in a similar manner and maintained at a constant thickness of ≈55 µm.

We initially evaluated the uniformity of the X‐ray energy deposition throughout the P3HSe: PC_70_BM, NP‐BHJ, and P3HT: PC_70_BM blends using FLUKA simulations (Figure S1, Supporting Information and Experimental Methods). Based on the FLUKA simulations for a 38 keV X‐ray (point) source, a total energy of 4.5 × 10^−4^ GeV cm^−3^ is deposited in the NP‐BHJ blend (Figure 1d) due to the higher X‐ray stopping power arising from the presence of Bi atoms. In comparison, an energy of 1.1 × 10^−4^ GeV cm^−3^ is deposited in the P3HSe: PC_70_BM blend film which is ≈25% of that observed for the NP‐BHJ detectors (Figure 1e). On the other hand, the energy deposition in the reference P3HT: PC_70_BM blend film was 2.8 × 10^−5^ GeV cm^−3^ (Figure S2, Supporting Information) which is attributed to the low X‐ray stopping power arising from the absence of high Z atoms. These results are in agreement with the bulk attenuation coefficient data (Figure S3, Supporting Information) as calculated using the NIST XCOM: Photon Cross Sections Database^[^ ^25^ ^]^ where the P3HT: PC_70_BM: Bi_2_O_3_ blend results in a higher mass attenuation coefficient of ≈9.7 cm^2^ g^−1^ compared to that of the P3HSe: PC_70_BM blend which has a mass attenuation coefficient of ≈3.2 cm^2^ g^−1^ at an X‐ray energy of 38 keV. However, we note that the electron‐hole pair (EHP) creation energy (*W*_±_) of the P3HSe: PC_70_BM, P3HT: PC_70_BM: Bi_2_O_3_, and the P3HT: PC_70_BM blends as calculated based on the Klein rule^[^ ^26^ ^]^ (Note S1, Supporting Information) indicates a much smaller *W*_±_ of 4.98 eV for P3HSe: PC_70_BM system as opposed to the P3HT: PC_70_BM: Bi_2_O_3_ system (*W*_±_ = 8.34 eV) and the P3HT: PC_70_BM system (*W*_±_ = 6.1 eV). Therefore, although P3HT: PC_70_BM: Bi_2_O_3_ blend system has a slightly higher energy deposition (as seen from FLUKA simulations), the P3HSe: PC_70_BM blend is hypothesised to produce nearly twice the amount of EHPs owing to its lower *W*_±_.

While energy deposition is widely modelled based on bulk mechanisms, the resulting charge generation processes of the materials have been shown to be dependent on the dimensionality of the materials used. For example, Guo and colleagues^[^ ^27^ ^]^ have extensively discussed the important role of nanoscale structures (Figure S4, Supporting Information) on the primary photoelectron generation process as a result of X‐ray absorption. These primary electrons deposit energy to the surrounding medium via excitation, ionization, and radiative losses.^[^ ^28^ ^]^ The generation of these primary electrons has been theoretically predicted to be more efficient when the dimensions of the high – Z material are reduced.^[^ ^29^ ^]^ These primary electrons in turn have been identified to lead to physical, chemical, and biological enhancement effects.^[^ ^27^ ^]^ The physical enhancement processes identified by Guo and colleagues indicate the generation of more energetic photoelectrons in X‐ray attenuating nanoparticles upon approaching dimensions of a few nanometers.^[^ ^27^ ^]^ The energy of these primary photoelectrons can also be dissipated through interactions with any oxygen atoms in the vicinity (referred to as chemical enhancement) and biological species (referred to as biological enhancement).^[^ ^27^ ^]^ Based on the enhancement processes discussed above, we identified the following key mechanisms for the X‐ray absorbers used in this study:

I) The primary electrons generated via photoelectric interaction with atomic Se in P3HSe are expected to result in more energetic primary electrons (Figure 1g), whereas most of the primary electrons generated from Bi atoms in Bi_2_O_3_ are likely to dissipate their energy upon interacting with the oxygen atoms (in the Bi_2_O_3_) (Figure 1f).

II) As the primary electrons are generated mainly within a Se atom in P3HSe, the resulting primary electrons can easily be transferred to PC_70_BM with little loss in energy resulting in more efficient hot carrier generation process as predicted by Thirimanne et al.^[^ ^11^ ^]^ In comparison, the energy loss that takes place from the primary electrons generated within Bi_2_O_3_ may result in a less efficient hot carrier generation process.

III) The recombination probability of an X‐ray generated hole and electron in the case of P3HSe is significantly reduced due to the shorter distance an electron must travel prior to being transferred to PC_70_BM (Note S2, Supporting Information). On the other hand, the “larger” dimensions of a Bi_2_O_3_ NP (in comparison to a Se atom) can result in the recombination of electrons and holes prior to transfer to the PC_70_BM and P3HT, respectively.

**Figure 2:**
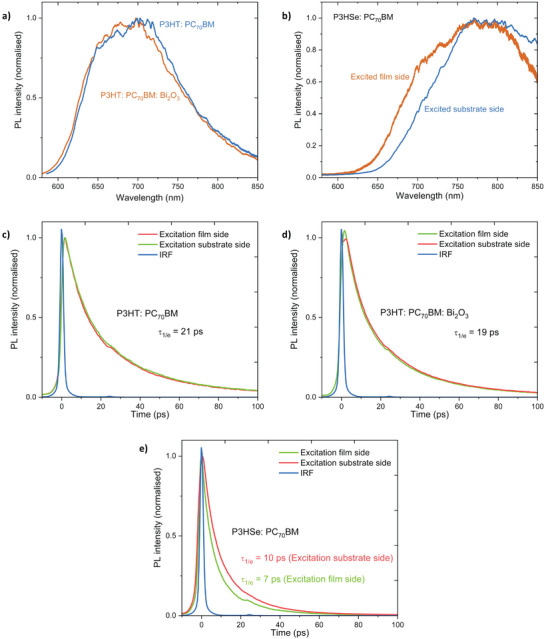
Charge transfer dynamics within the films. a) PL spectra of the P3HT: PC_70_BM and P3HT: PC_70_BM: Bi_2_O_3_ blend films which indicate that the insertion of NPs has no significant effect on aggregation of conjugated P3HT chains. b) PL spectra of the P3HSe: PC_70_BM blend film which indicate slightly different characteristics when excited from the substrate and film sides. PL decays of the c) P3HT: PC_70_BM, d) P3HT: PC_70_BM: Bi_2_O_3_, and e) P3HSe: PC_70_BM blend films. The faster PL decay when excited from the film side in P3HSe: PC_70_BM blend film indicates that the average domain sizes are smaller at the surface of the film.

While the direct observation of mechanisms I) and II) remain challenging, mechanism III) can be evaluated based on transient optical spectroscopy. Therefore, we conducted steady‐state photoluminescence (PL) spectra and time resolved photoluminescence (TRPL) measurements to analyze the charge transfer dynamics of the systems studied here. For the P3HT: PC_70_BM blend, (**Figure** **2**a) steady–state PL measurements indicated a broad emission from 650 to 720 nm that is characteristic of semi‐crystalline P3HT.^[^ ^30^, ^31^ ^]^ The NP‐BHJ films also displayed a similar steady state PL spectrum, suggesting that insertion of NPs has no significant effect on the aggregation of conjugated P3HT chains (Figure 2a). To further identify the charge transfer processes that take place within these films and to explore their morphology, we conducted TRPL measurements. In blends with PC_70_BM, the photoluminescence of P3HT is quenched in the event of an efficient electron transfer or energy transfer to the fullerene derivative.^[^ ^30^ ^]^ The quenching rate is limited by the time it takes for the exciton in P3HT or P3HSe to diffuse to an interface with PC_70_BM with smaller semiconducting polymer domains resulting in faster PL quenching.^[^ ^32^ ^]^ As can be seen from Figure 2c and d, NP‐BHJ films displayed slightly faster PL quenching compared to the P3HT: PC_70_BM blend films indicative of the formation of slightly smaller P3HT domains upon the insertion of Bi_2_O_3_ NPs. To evaluate the associated time scales, we determined $\tau_{1/e}$ which is defined as the time for the PL intensity to decay to 1/e of its initial value. For the NP‐BHJ films, this was estimated to be 19  ±  1 ps as opposed to 21  ±  1 ps for the P3HT: PC_70_BM blend films. These results agree with the $\tau_{1/e}$ value reported by Ruseckas et al.^[^ ^32^ ^]^ for the 50: 50 wt.% P3HT: PC_60_BM blends where a decay time of 22  ±  1 ps was observed. Furthermore, Ruseckas et al.^[^ ^32^ ^]^ also estimated an average PC_70_BM domain size (*d*) of 9 nm based on:

(1) $$d\approx 2\sqrt{3fD\tau_{1/e}}$$

where *f* is the fraction of PC_70_BM in the blend (by mass) and *D* is the exciton diffusion coefficient in P3HT. Based on the above, a (similar) domain size of ≈ 9 nm is estimated for PC_70_BM in the P3HT: PC_70_BM blend films assuming spherical shape of these domains. We note that in reality the domains are irregular and not uniform, hence, this number is only an indicator of the average domain size. Slightly faster PL decay observed with the addition of Bi_2_O_3_ NPs suggests that the average domain size decreases by ≈1 nm or the domain shape becomes more irregular. In any case these changes are small and insignificant as the PL spectra and PL decays showed no discrepancy when excited from film side and substrate side despite enrichment of Bi_2_O_3_ at the bottom. Furthermore, NP‐BHJ films displayed similar PL decay times when excited from the substrate and film sides, suggesting that the average domain sizes are uniform throughout a large portion of the film (Figure 2d).

In comparison, films based on P3HSe: PC_70_BM show slightly different PL spectra and PL decays when excited from the substrate and film sides (Figure 2b and e). In the P3HSe: PC_70_BM film, excitation from the film side shows an additional shoulder in the PL spectrum at shorter wavelengths ≈700 nm and a faster decay ($\tau_{1/e}$ = 7  ±  1 ps) as compared to excitation from the substrate side ($\tau_{1/e}$ = 10  ±  1 ps) (Figure 2e). The faster PL decay when excited from the film side indicates that the average domain size is smaller at the surface of the film. The shape of P3HSe PL spectrum resembles that of P3HT which is known to form spectroscopic H aggregates where the 0‐0 vibronic peak at shorter wavelengths (visible as a shoulder when excited from the film side in Figure 2b) indicates increased disorder.^[^ ^33^ ^]^ Hence, the PL shoulder at shorter wavelengths suggests that the P3HSe film is more amorphous at the surface. Since an amorphous P3HSe mixes better with PC_70_BM, this explains the smaller domain sizes at the film surface.^[^ ^34^ ^]^ Since P3HSe PL has a very small spectral overlap with PC_70_BM absorption (and therefore energy transfer from P3HSe to PC_70_BM cannot take place), the electron in P3HSe has to diffuse very close to the interface with PC_70_BM for PL quenching to occur. In this situation, *d* is estimated using the Equation (1) where *D* is the exciton diffusion coefficient in P3HSe. Assuming that *D* is similar to that of P3HT, then the observed $\tau_{1/e}$ indicates domain sizes of ≈5 nm (on the film side) and 6 nm (on the substrate side) in P3HSe: PC_70_BM blends.

**Figure 3:**
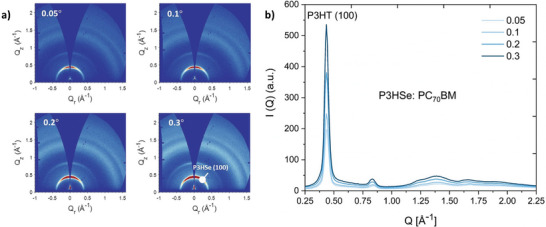
Crystallinity of the films. a) 2D GIWAXS spectra obtained at 0.05°, 0.1°, 0.2°, and 0.3° incident angles for the P3HSe: PC_70_BM blend film indicating the increase of the (100) peak signal at *Q_z_* ≃ 0.4 Å^–1^ with depth. b) 1D azimuthally integrated intensity profiles of the P3HSe: PC_70_BM blend film across the full azimuthal range for various incidence angles.

In order to directly quantify the changes in the domain sizes for P3HT and P3HSe through the thickness of the film, we carried out grazing incidence wide angle X‐ray scattering (GIWAXS) (**Figure** **3**; Figure S5, Supporting Information). This technique enables probing of the sub‐surface as well as the “bulk” of the blend films up to ≈2 µm depth by changing the incident angle of the X‐ray beam. For the P3HT: PC_70_BM and NP‐BHJ films, we observed a narrow peak at *Q_z_* ≃ 0.4 Å^–1^ corresponding to the (100) lamellar stacking of P3HT polymeric chains, indicating the crystallization of the P3HT phase across the examined film depth (Figure S5, Supporting Information). In a similar manner, P3HSe: PC_70_BM blend films also displayed a narrow peak at *Q_z_* ≃ 0.4 Å^–1^, suggesting that P3HSe polymeric chains have also adopted a (100) lamellar stacking, thus indicating crystallization of the P3HSe phase^[^ ^35^ ^]^ (Figure 3a). From the 1D intensity profiles extracted in the out‐of‐plane and in‐plane directions, the higher intensity of the P3HT (100) and PSHSe (100) peaks in the out‐of‐plane direction (i.e., the *Q_z_* direction, normal direction to the substrate) compared to the in‐plane direction are characteristic of a predominantly edge‐on orientation (Figure S6, supporting information). In this configuration, π‐π stacking is in‐plane (perpendicular to the substrate) and lamellar stacking is in the out‐of‐plane direction (parallel to the substrate).^[^ ^36^ ^]^

Using the Scherrer formula (Note S3, Supporting Information), we estimated the crystalline coherence length (CCL) (Table S2, Supporting Information) for the p‐type polymers for the blends studied in this work. For the NP‐BHJ blend films, a slightly smaller P3HT CCL of 16 ± 0.4 nm was estimated compared to the P3HT: PC_70_BM blend where a CCL of 17 ± 0.4 nm was estimated. Moreover, the P3HSe: PC_70_BM blend films displayed even smaller CCL of 13 ± 0.3 nm, which is in good agreement with the faster PL decay characteristics and the blue shifted steady state PL spectra. Furthermore, each blend film displayed an increase in the CCL with increasing incidence angle (Table S2, Supporting Information). This observation agrees with the characteristics evident from the PL spectra for the P3HSe: PC_70_BM blend films where a reduction of the crystallinity of the P3HSe domains was observed as one moves toward the film surface away from the bulk.

### 2.2 Response Characteristics of The Detectors

**Figure 4:**
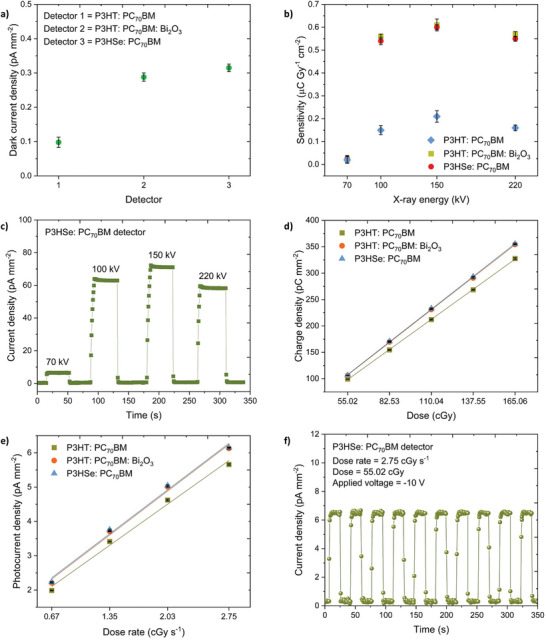
Response characteristics of the detectors. a) Comparison of dark current response of the detectors based on P3HT: PC_70_BM, P3HT: PC_70_BM: Bi_2_O_3_, and P3HSe: PC_70_BM blend films under an applied bias of ‐10 V. b) Comparison of sensitivity of the detectors based on P3HT: PC_70_BM, P3HT: PC_70_BM: Bi_2_O_3_, and P3HSe: PC_70_BM blend films under the 70, 100, 150, and 220 kVp X‐ray radiation. c) Transient X‐ray photocurrent response from the P3HSe: PC_70_BM detectors under different X‐ray energies indicating a “square shaped” response. d) Dose linearity and e) Dose rate linearity of the X‐ray detectors based on P3HT: PC_70_BM, P3HT: PC_70_BM: Bi_2_O_3_, and P3HSe: PC_70_BM blend films under the 70 kVp X‐ray radiation. The solid lines indicate linear fits. Both the dose as well as dose rate dependence show excellent linearity (R^2^ > 0.9998). f) Reproducibility of the photocurrent response of the X‐ray detectors based on P3HSe: PC_70_BM blend film under the 70 kVp X‐ray radiation. The data points in the Figure a), b), d), and e) are averaged over three separate detector measurements.

Initially, we studied the dark diode characteristics of the detectors. Having a low dark current is of paramount importance for high quality X‐ray detection, since the dark current determines the limit of detection, contributing signal‐to‐noise ratio, and the dynamic range, which are crucial parameters in dosimetry and medical imaging.^[^ ^15^ ^]^ As can be seen from **Figure** **4**a, the detectors based on P3HSe: PC_70_BM demonstrated an ultra‐low dark current of 0.32 ± 0.01 pA mm^−2^ under an applied bias of −10 V. This value is well within the industrial standard of 10 pA mm^−2[^ ^37^ ^]^ and is also comparable to the dark current observed for the NP‐BHJ X‐ray detectors (0.28 ± 0.01 pA mm^−2^). However, the reference detector which is based on the P3HT: PC_70_BM blend displayed an even lower dark current response of 0.1 ± 0.02 pA mm^−2^. The presence of high – Z atoms in the P3HSe: PC_70_BM and NP‐BHJ blend creates more defect states that can lead to the formation of stray charge carriers^[^ ^38^ ^]^ whereas the absence of high – Z atoms in the reference matrix leads to fewer stray charge carriers, hence the lower dark current response. It should also be noted that the P3HSe: PC_70_BM and NP‐BHJ X‐ray detectors display the lowest dark currents reported so far compared to the organic, hybrid, and perovskite detectors in the literature (Figure S7, Supporting Information). In addition, the P3HSe: PC_70_BM detectors demonstrated a rise in dark current up to 7.6 pA mm^−2^ with increasing applied bias from −10 to −200 V (i.e., equivalent to a bulk electric field from 0.2 to ≈4 V µm^−1^) which is very similar to that of the NP‐BHJ X‐ray detectors which rose up to 6.9 pA mm^−2^ at −200 V (Figure S8, Supporting Information).

Following dark diode characterization, we compared the photocurrent response characteristics of the detectors under X‐ray radiation. Since the effective Z of the P3HSe: PC_70_BM blend and the NP‐BHJ blend are different, the response characteristics of the detectors are anticipated to change based on the X‐ray energy / tube voltage. Therefore, to study the effect of the X‐ray energy / tube voltage on the detector response, we evaluated the sensitivity of the detectors under 70, 100, 150, and 220 kVp X‐ray radiation where the detector sensitivity is estimated based on the equation given below:^[^ ^14^ ^]^

(2) $$S=\frac{Q}{DA}=\frac{\int \left(I_{ON}\left(t\right)-I_{OFF}\right)dt}{DA}$$

where, Q is the charge generated under X‐ray irradiation, *I_ON_* and *I_OFF_* are the current generated with and without X‐ray exposure, respectively, D is the X‐ray incident dose, and A is the active area of the detector. When exposed to X‐rays from the 70, 100, 150, and 220 kVp X‐ray sources, the detectors based on P3HSe: PC_70_BM exhibited sensitivities of 22.6 ± 1, 540 ± 16, 600 ± 11, and 550 ± 11 nC Gy^−1^ cm^−2^, respectively. These values are comparable to the sensitivities observed from NP‐BHJ X‐ray detectors (22.4 ± 2, 560 ± 10, 610 ± 26, and 570 ± 12 nC Gy^−1^ cm^−2^, respectively) (Figure 4b). In comparison to the above detectors, the reference detector based on P3HT: PC_70_BM displayed a slightly lower sensitivity values (20.7 ± 1, 150 ± 12, 210 ± 20, and 160 ± 15 nC Gy^−1^ cm^−2^, respectively) (Figure 4b) which can be explained by the absence of high – Z atoms within the reference detector matrix. This increase in sensitivity with X‐ray energy can be attributed to the high X‐ray energy deposition leading to the generation of more charge carriers. We also note that high Z elements incorporated at the atomic level can lead to enhancement effects as reported for example by Ting Guo's group^[^ ^27^ ^]^ which leads to more X‐ray energy deposition resulting in the generation of more charge carriers. A comparison of the sensitivity observed from the P3HSe: PC_70_BM and NP‐BHJ X‐ray detectors with the sensitivities reported in the literature for stabilized amorphous selenium, cadmium zinc telluride, organic, hybrid, and perovskite detectors are shown in Figure S10 (Supporting Information). Despite the relatively low thickness of these absorbers, P3HSe: PC_70_BM and NP‐BHJ detectors display a satisfactory performance compared to more established, state‐of‐the‐art detector technologies. Furthermore, the X‐ray response of the NP‐BHJ and P3HSe: PC_70_BM X‐ray detectors, display a “square shaped” X‐ray photocurrent response with a sharp rise and decay (Figure 4c; Figure S11, Supporting Information). This is contrary to the saw‐tooth shaped transient photocurrent response reported for a number of organic semiconductor‐based X‐ray detectors^[^ ^39^, ^40^ ^]^ or for previous generation of NP‐BHJ detectors that use PEDOT: PSS as the HTL in combination with a regular device architecture.^[^ ^11^ ^]^ The observation of saw‐tooth shaped characteristics has been attributed to the photoconductive gain mechanisms in mono‐carrier type devices.^[^ ^39^ ^]^ The lack of such a characteristic in the NP‐BHJ and P3HSe: PC_70_BM X‐ray detectors developed here suggests a more balanced, X‐ray generated electron and hole extraction mechanism to be prevalent in these devices. From an X‐ray attenuation point of view, the lower mass attenuation coefficient of P3HSe: PC_70_BM compared to the NP‐BHJ absorber is expected to result in a lower X‐ray sensitivity. However as shown previously, the atomic nature of the absorber in P3HSe as opposed to the larger particle like nature in Bi_2_O_3_ results in a more efficient charge transfer process which together with the higher Schubweg, and higher charge carrier generation (explained later in the discussion) compensate for the lower X‐ray attenuation in the former system and lead to the similar sensitivities observed for both type of detectors. Furthermore, the P3HSe: PC_70_BM detectors also displayed excellent dose and dose rate linearity (Figure 4d,e), as well as high reproducibility under repeated X‐ray exposures (Figure 4f; Figure S12, Supporting Information) similar to NP‐BHJ detectors, indicating the promise of heteroatom substitution in organic semiconductors for X‐ray detection.

Furthermore, long term stability under storage as well as under repeated X‐ray exposures is a requirement of a semiconducting material for X‐ray detection. Therefore, the stability of the detector characteristics was evaluated by measuring the dark current and X‐ray photocurrent response of a detector (stored in a N_2_ environment in the dark in‐between measurements) over a 12‐month period (Figure S13, Supporting Information). Although a slight increase in the dark current density was observed, the dark current density remained less than 1 pA mm^−2^ (at −10 V), well within industrially accepted values. This is indicative of the inherent stability of this device architecture in comparison to other emerging detector systems such as perovskites which can suffer from delamination from the substrate over longer time periods.^[^ ^41^ ^]^ The X‐ray photocurrent response as evaluated under a 70 kV X‐ray source also did not show a noticeable variation (Figure S13, Supporting Information). The good stability observed for both dark current and X‐ray photocurrent indicates the excellent stability of the P3HSe: PC_70_BM system. To further evaluate the stability of the detectors under repeated exposures, the detectors were subject to a series of repeated X‐ray exposures under a 70 kV X‐ray source until a cumulative dose of 100 Gy was achieved. As can be seen from Figure S13 (Supporting Information), the detectors did not display any significant degradation of performance thus highlighting the excellent radiation hardness of the P3HSe: PC_70_BM system.

### 2.3 Charge Transport Processes

**Figure 5:**
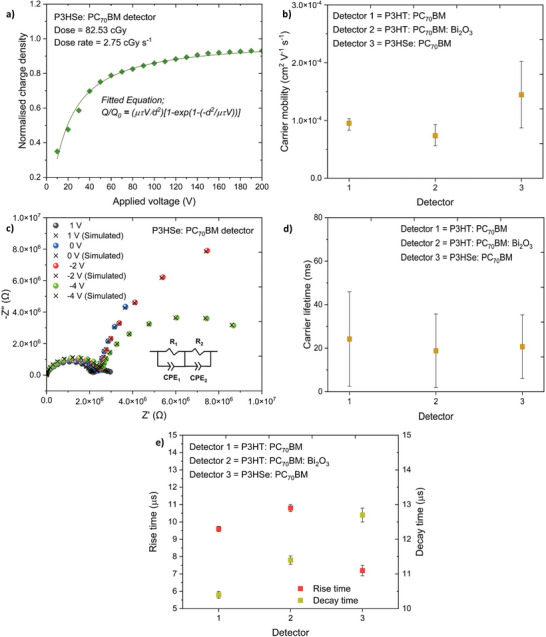
Charge transport characteristics of the detectors. a) Voltage dependence together with the Hecht fit (R^2^ > 0.9998) of the X‐ray detectors based on P3HSe: PC_70_BM blend film. b) Comparison of charge carrier mobility estimated from the photo‐CELIV method of the detectors based on P3HT: PC_70_BM, P3HT: PC_70_BM: Bi_2_O_3_, and P3HSe: PC_70_BM blend films. c) Nyquist plot of the P3HSe: PC_70_BM based detector under dark conditions when biased at +1, 0, −2, ‐4 V. The black colour crosses (×) represent the fits for each bias calculated using the equivalent circuit shown in the inset. R_1_ and R_2_ are resistance components forming a parallel circuit with the constant phase elements CPE_1_ and CPE_2_. d) Comparison of charge carrier lifetime estimated from IS of the detectors based on P3HT: PC_70_BM, P3HT: PC_70_BM: Bi_2_O_3_, and P3HSe: PC_70_BM blend films. e) Comparison of rise time and decay times estimated from the TPC method for the detectors based on P3HT: PC_70_BM, P3HT: PC_70_BM: Bi_2_O_3_, and P3HSe: PC_70_BM blend films. The data points in Figure b), d), and e) are averaged over three separate detector measurements.

The mobility‐lifetime (μτ) product is a key parameter for X‐ray detection which is typically assessed using the Hecht equation given below^[^ ^40^ ^]^:

(3) $$Q=Q_{0}\;\frac{\mu \tau V}{d^{2}}\left[1-exp\left(-\frac{d^{2}}{\mu \tau V}\right)\right]$$

where *Q* is the total extracted charge, *Q*_0_ is the asymptotic charge, *V* is the applied voltage, *d* is the detector active layer thickness, μ is the carrier mobility, and τ is the carrier lifetime. By analyzing the voltage dependence of X‐ray photocurrent response over the voltage range of −10 to −200 V (**Figure** **5**a; Figure S14, Supporting Information), we estimated a μτ product of 3 × 10^−6^ ± 5 × 10^−8^ cm^2^ V^−1^ for the P3HSe: PC_70_BM detectors which is slightly higher compared to that observed from the NP‐BHJ X‐ray detectors (1.4 × 10^−6^ ± 2 × 10^−8^ cm^2^ V^−1^).

To form a better understanding of the origins of the high μτ product, we carried out electronic characterization of the X‐ray absorber blends. Based on the photo charge extraction by linearly increasing voltage (photo‐CELIV) analysis, we observe a charge carrier mobility value of 1.4 × 10^−4^ ± 5.8 × 10^−5^ cm^2^ V^−1^ s^−1^ for the P3HSe: PC_70_BM detectors which is nearly a factor of two higher than the mobility values observed from the NP‐BHJ X‐ray detector (7.4 × 10^−5^ ± 1.9 × 10^−5^ cm^2^ V^−1^ s^−1^) and the P3HT: PC_70_BM reference detector (9.5 × 10^−5^ ± 7.7 × 10^−6^ cm^2^ V^−1^ s^−1^) (Figure 5b). The charge carrier lifetimes within the different absorbers were also estimated based on impedance spectroscopy (IS) measurements under dark conditions. The impedance spectra were acquired under four applied voltage conditions (+1, 0, −2, ‐4 V) (Figure 5c; Figure S15, Supporting Information).^[^ ^42^ ^]^ A lifetime of 20.7 ± 15 ms was calculated for the P3HSe: PC_70_BM based detectors while a lifetime of 18.8 ± 17 ms was estimated for the NP‐BHJ detectors (Figure 5d). The μτ products estimated independently from the photo‐CELIV (for carrier mobility) and IS (for carrier lifetime) are in good agreement with the μτ product values estimated from the voltage dependence studies.

Following the estimation of charge carrier mobility and charge carrier lifetime, we estimated the Schubweg ($\overset{`}{S}$) which indicates the charge carrier drift length or the distance a carrier drifts before being deeply trapped and becoming unavailable for conduction^[^ ^43^ ^]^ using:

(4) $$\overset{`}{S}=\mu \tau E$$

Here, μ is the charge carrier mobility, τ is the carrier lifetime, and *E* is the applied electric field. Using the carrier mobility and carrier lifetime values estimated using photo‐CELIV and IS measurements and the applied macroscopic electric field to be 0.18 × 10^4^ V cm^−1^ (which corresponds to an applied bias of −10 V), a Schubweg of 25 ± 6 µm is estimated for the P3HT: PC_70_BM: Bi_2_O_3_ system. Since the NP‐BHJ film is ≈55 µm thick and a majority of Bi_2_O_3_ NPs are enriched toward the bottom half of the film, having such a small Schubweg value indicates that most of the charge carriers are likely to be trapped prior to collection, resulting in a reduction in the charge collection efficiency. In comparison, a relatively high Schubweg value of 53 ± 16 µm was estimated for the P3HSe: PC_70_BM system which indicates a more efficient carrier extraction process for this system.

In order to evaluate the rise and decay time constants which directly influence the time response characteristics (or the lag and ghosting behavior^[^ ^44^ ^]^) of X‐ray detectors, we conducted transient photocurrent (TPC) measurements. The P3HSe: PC_70_BM detectors demonstrated a relatively fast rise time of 7.2 ± 0.3 µs (Figure 5e) in comparison to rise times of 10.8 ± 0.2 and 9.6 ± 0.2 µs obtained for the NP‐BHJ and P3HT: PC_70_BM detectors, respectively. On the other hand, P3HSe: PC_70_BM detectors and the NP‐BHJ detectors demonstrated decay time constants of 12.7 ± 0.2 and 11.4 ± 0.1 µs, respectively (Figure 5e). Such relatively fast response characteristics of the P3HSe: PC_70_BM detectors are in good agreement with the higher charge transfer and charge carrier mobilities observed, thereby leading to better charge extraction. The low decay time constants of less than 15 µs for the P3HSe: PC_70_BM based detectors and the NP‐BHJ detectors suggest their suitability for real‐time image acquisition such as computed tomography where low rise and decay times are desirable for reducing lag and ghosting effects.^[^ ^44^ ^]^

### 2.4 Effect of Heteroatom Inclusion on Detector Performance

For a direct X‐ray detector such as those used in this study, the sensitivity is influenced by three key steps: X‐ray absorption, charge pair creation, and charge transport and collection.^[^ ^15^ ^]^ Kasap and co‐workers have reported a definition for sensitivity (*S*)^[^ ^37^ ^]^ based on the efficiency of three stages as given below:

(5) $$S=S_{o}\;\eta_{x}\eta_{m}\eta_{cc}$$

Here, *S_o_* is an X‐ray energy dependent constant, η_*x*_ is the quantum efficiency, η_*m*_ is the number of EHPs created per absorbed photon, and η_*cc*_ is the charge collection efficiency. The above expression suggests that the sensitivity of a detector can be increased by maximizing the product η_*x*_ × η_*m*_ × η_*cc*_. This can be achieved by using an X‐ray absorber that possesses higher X‐ray energy deposition (i.e., high quantum efficiency), low electron‐hole pair creation energy (i.e., higher number of generated charge pairs), and high charge collection efficiency.

Based on the bulk attenuation coefficient data and the X‐ray energy deposition simulations, it is evident that the NP‐BHJ system can offer a higher X‐ray attenuation (i.e., higher η_*x*_). However, it was also noticed that the P3HSe: PC_70_BM system can generate nearly twice the amount of EHPs compared to the NP‐BHJ system owing to its low *W*_±_ (i.e., higher η_*m*_). The TRPL measurements also demonstrated that the charge transfer dynamics are faster in the P3HSe: PC_70_BM system compared to the NP‐BHJ system which is attributable to the smaller P3HSe domain sizes. Moreover, the relatively high charge carrier mobility as well as the mobility‐lifetime product of the P3HSe: PC_70_BM system was identified to result in a relatively high Schubweg of 53 ± 16 µm which enables most of the carriers to traverse across the 55 µm thick film without being trapped. As explained earlier, incorporating high – Z Se heteroatoms in the organic semiconductor structure prompts uniform distribution of high – Z atoms across the active layer whereas the inclusion of high – Z NPs leads to the enrichment toward the bottom of the film. This agglomeration of NPs toward the bottom of the film combined with the lower Schubweg of 25 ± 6 µm may result in some of the charge carriers generated within the 55 µm thick NP‐BHJ film being trapped before reaching the electrode. Based on these observations, it is proposed that the P3HSe: PC_70_BM system possesses better charge transfer and transport characteristics (i.e., higher η_*cc*_). Hence, according to the definition of theoretical sensitivity (Equation (5)) which highlights maximizing the η_*x*_ × η_*m*_ × η_*cc*_ product, the above results justify the comparable performance observed from the NP‐BHJ X‐ray detectors and the P3HSe: PC_70_BM X‐ray detectors.

### 2.5 Fabrication of Curved X‐Ray Detectors

In order to fabricate curved X‐ray detectors based on the P3HSe: PC_70_BM blend, an understanding of the compatibility between the P3HSe: PC_70_BM blend film and the flexible substrate is required. Based on the insights developed for the NP‐BHJ system in our previous study,^[^ ^6^ ^]^ polyimide was used as the flexible substrate in this study. To evaluate the effect of the P3HSe: PC_70_BM blend on the compatibility between the film and the substrate, nano‐mechanical properties (Young's modulus and hardness) of the P3HSe: PC_70_BM film were studied by conducting nano‐indentation analysis.^[^ ^6^ ^]^ The mechanical properties at the film surface are estimated by analyzing the measured load–displacement curves (*P*–*h* curves). Figure S16 (Supporting Information) illustrates the loading and unloading *P* − *h* curves for the P3HSe: PC_70_BM film and NP‐BHJ film. The slope for both the loading and unloading curves (shown as dashed lines here) are comparable for both films, indicating the similarity in the ordering of the blend morphology and thus the nano‐mechanical properties.

**Figure 6:**
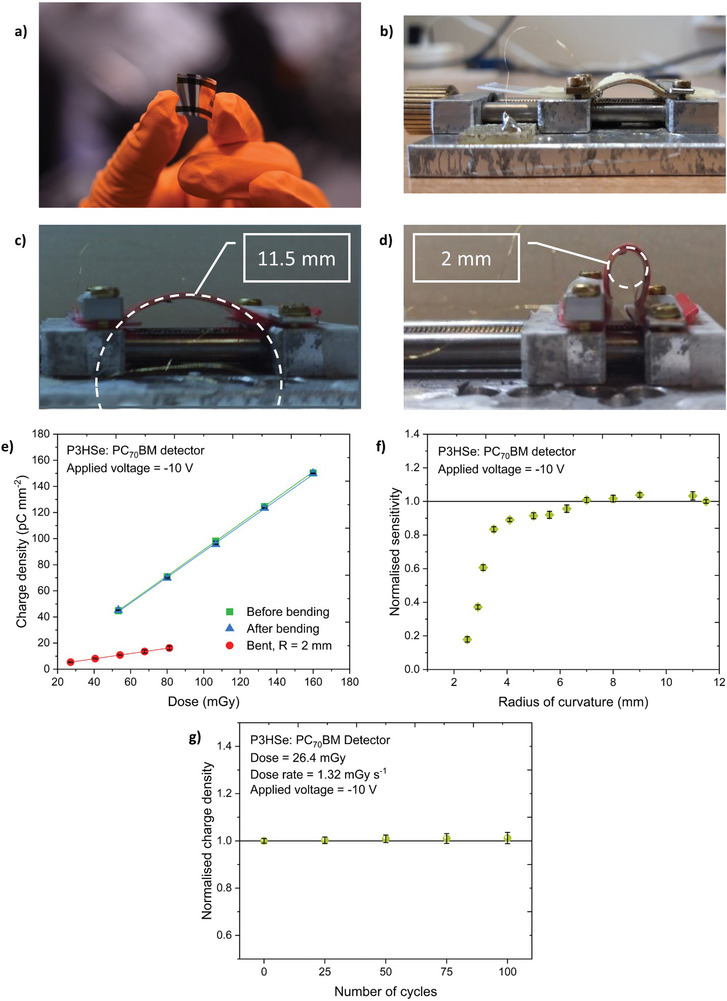
Bendability characteristics of the curved detectors. a) Photograph of a curved P3HSe: PC_70_BM X‐ray detector, b) Experimental set‐up used for the bendability characterization of the hybrid detectors, Photograph of a curved P3HSe: PC_70_BM X‐ray detector bent to a radius of curvature of c) 11.5 mm and d) 2 mm. e) Charge density as a function of the incident dose for the P3HSe: PC_70_BM detector measured before bending (green solid squares), during bending with a bending radius of 2 mm (red solid circles), and after bending (blue solid triangles) indicating dose linearity under each condition, f) Normalized sensitivity of the P3HSe: PC_70_BM detector as a function of bending radius indicating the threshold curvature radius limit for bendability, g) Normalized charge density of the P3HSe: PC_70_BM detector measured before bending, and after 25, 50, 75, and 100 bending cycles. The data points in Figure e), f), and g) are averaged over three separate detector measurements.

The Young's modulus, *E* (Note S4, Supporting Information) estimated for the P3HSe: PC_70_BM film (6.3 ± 0.1 GPa) is shown to be comparable to that of the NP‐BHJ film (5.9 ± 0.2 GPa). Since the stiffness of a material is proportional to the *E*, this indicates that both films have a similar stiffness. Furthermore, the hardness (Note S5, Supporting Information) for both P3HSe: PC_70_BM and NP‐BHJ films displayed similar values of 157 ± 11 and 154 ± 15 MPa, respectively. Based on the similarity of the nano‐mechanical properties of the films, it can be expected that both films display similar misfit strain, or compatibility with the flexible substrate. Based on our previous study^[^ ^6^ ^]^ where the misfit strain was lower when thicker polyimide substrates are used in combination with NP‐BHJ films, it can be deduced that employing thicker polyimide substrates would be practicable for the fabrication of curved detectors based on P3HSe: PC_70_BM films. Therefore, curved X‐ray detectors (**Figure** **6**a) were fabricated on 75 µm thick polyimide films with a device layer stacking of polyimide/ Al/ Al‐doped ZnO/ P3HSe: PC_70_BM blend/ NiO/ Ag.

To assess the detector response under different bending radii, the measurement setup shown in Figure 6b was used in conjunction with an X‐ray source operating at 40 kVp. The detector was fixed between two PET substrates which are then clamped to the measurement setup to assure that the detector active area is fully subjected to the bending radius. It should be noted that the detectors already demonstrated a natural radius of 11.5 mm in its pristine condition (Figure 6c) due to the inherent misfit strain between the P3HSe: PC_70_BM layer and the polyimide substrate. The P3HSe: PC_70_BM detectors irradiated under this initial condition displayed a square shaped, reproducible photocurrent response with an X‐ray generated photo‐charge magnitude that is linearly increasing with incident dose. Based on the dose linearity response, a sensitivity value of 0.1 ± 0.004 µC Gy^−1^ cm^−2^ was observed for the P3HSe: PC_70_BM detector alongside a dark current response as low as 0.03 ± 0.01 pA mm^−2^ when biased at −10 V. The sensitivity of the P3HSe: PC_70_BM X‐ray detectors was compared with the sensitivities reported in the literature for other flexible organic, perovskite, and our previous detectors as shown in Figure S17 (Supporting Information). Despite the relatively low thickness of this absorber, P3HSe: PC_70_BM detectors display a satisfactory sensitivity compared to other detector technologies. Furthermore, these detectors possess additional benefits of tissue equivalence and conformable functionality which make them ideal candidates for radiotherapy and medical dosimetry applications. In fact, such detectors might be interposed between the X‐ray source and the patient enabling real‐time in situ dosimetry, without significantly absorbing the incoming X‐ray beam. It should be noted that there are currently no low‐cost, large‐area detectors with tissue equivalence and conformable functionality available.

The detectors displayed excellent resistance to mechanical failure, even when bent to a radius as small as 2 mm (Figure 6d). This implies that these detectors are also conformable to most of the curved surfaces indicating their potential use in a wide range of applications such as medical imaging and diagnostics.

Initially, the performance of curved detectors based on P3HSe: PC_70_BM and the NP‐BHJ blend under 40 kVp X‐ray radiation was compared before bending, while bent (to a radius of 2 mm), and after bending (i.e., relaxation to its initial state). The resulting detector sensitivity values are shown in Figure 6e and Figure S18 (Supporting Information). The sensitivity of the detectors based on P3HSe: PC_70_BM blend appeared to decrease during bending where a ≈20% reduction in the sensitivity was observed (compared to an initial sensitivity of 0.1 ± 0.004 µC Gy^−1^ cm^−2^). On the other hand, the sensitivity of the NP‐BHJ X‐ray detectors improved by 170% during bending compared to an initial sensitivity value of 0.1 ± 0.004 µC Gy^−1^ cm^−2^. However, the performance of both detectors appeared to be almost recovered to its initial sensitivity after bending where a sensitivity of 0.1 ± 0.003 Gy^−1^ cm^−2^ was achieved.

Furthermore, a curved detector is required to display a consistent performance even when subjected to extreme bending conditions. Therefore, to compare the threshold curvature radius of bendability for the P3HSe: PC_70_BM and NP‐BHJ X‐ray detectors, the variation in the detector sensitivity under a series of bending radii (from 11.5 to 2 mm) was compared to the sensitivity in pristine condition (Figure 6f; Figure S19, Supporting Information). The sensitivity at each radius of curvature was normalized to that of the pristine condition. It was noticed that up to a threshold radius of 3.5 mm, the P3HSe: PC_70_BM detectors do not display any significant deviation of sensitivity, whereas beyond this threshold limit, the photocurrent reduces considerably in comparison to the NP‐BHJ system where such a reduction is not observed. This performance/sensitivity reduction beyond the threshold limit is attributed to the temporary increase of the chemical bond lengths within P3HSe which can also alter its electronic characteristics, thereby leading to less efficient charge transfer from P3HSe to PC_70_BM.^[^ ^45^ ^]^ On the other hand, the sensitivity of the NP‐BHJ detectors demonstrated significant enhancement beyond a threshold radius of 2.5 mm. As explained in our previous study,^[^ ^6^ ^]^ this performance/sensitivity enhancement beyond the threshold limit in NP‐BHJ detectors is attributed to a “temporary arrangement” within the NP‐BHJ blend where the aggregated NPs are separated from each other during deformation, thus leading to the resulting volume being occupied by the P3HT and PC_70_BM.

In addition, it is necessary for a curved detector to display reproducibility in performance when subjected to multiple bending cycles. Hence, the mechanical robustness of the P3HSe: PC_70_BM and NP‐BHJ detectors was compared by conducting dynamic bending up to 100 cycles down to a radius of 2 mm as shown in Figure 6g and Figure S20 (Supporting Information). The charge density (which is proportional to the sensitivity) of each detector after 25, 50, 75, and 100 bending cycles was normalized to the charge density (or the sensitivity) before bending. The detectors do not display a significant degradation upon repeated bending, with a maximum deviation of ≈1.2% and 3.2%, for charge density (or sensitivity) being observed for P3HSe: PC_70_BM and NP‐BHJ detectors, respectively.

## 3 Conclusion

We have developed a high‐performance organic X‐ray detector based on P3HSe: PC_70_BM blend film by modifying the organic semiconducting polymer chain with high – Z heteroatoms. When characterized under the 70, 100, 150, and 220 kVp X‐ray radiation, P3HSe: PC_70_BM detectors displayed a superior performance in terms of sensitivity up to 600 ± 11 nC Gy^−1^ cm^−2^. Based on complementary characterization techniques such as voltage dependence, photo‐CELIV, FLUKA energy deposition simulations, and TRPL, it was realized that employing organic semiconductors modified with high – Z heteroatoms enables a higher number of electron‐hole pairs (i.e., higher η_*m*_), better charge transfer and transport characteristics (i.e., higher η_*CC*_), which ultimately maximizes the η_*X*_ × η_*m*_ × η_*CC*_ product, thus resulting in high performance. Curved X‐ray detectors based on P3HSe: PC_70_BM also demonstrated mechanical flexibility up to a bending radius as small as 2 mm, and a stable detector performance with less than 1.2% variation in performance under 100 repeated bending cycles. Furthermore, favorable detector response characteristics such as an ultra‐low dark current response of 0.03 ± 0.01 pA mm^−2^ and a sensitivity value of 0.1 ± 0.004 µC Gy^−1^ cm^−2^ have also been observed from such curved detectors under bending. This study confirms the heteroatom incorporation as a successful strategy toward realizing X‐ray detectors based on organic semiconductors with broadband high sensitivity and ultra‐low dark current response while preserving the tissue equivalence and curved functionality. We believe this study will spark further interest in the academic and the industrial community toward employing heteroatom modified organic semiconductors for X‐ray detection in a range of demanding applications including medical imaging and radiotherapy dosimetry.

## 4 Experimental Section

### Materials

Regioregular poly(3‐hexylthiophene‐2,5‐diyl) (P3HT) of 4002‐EE grade was purchased from Rieke Metals. Regioregular (98%) poly(3‐hexyl)selenophene (P3HSe) was prepared as previously reported,^[^ ^17^ ^]^ with a weight average molecular weight (Mw) of 97,500 g mol^−1^ and a dispersity (Ð) of 1.29, as measured by gel permeation chromatography (chlorobenzene, 80 °C) against polystyrene standards. [6,6]‐phenyl C71 butyric acid methyl ester (PC_70_BM) of purity >99% was purchased from Solenne. Bismuth oxide nanoparticles (Bi_2_O_3_) (with a β phase, tetragonal crystal structure; 38 nm diameter; surface area 18 m^2^ g^−1^) were purchased from Alfa Aesar. The P3HT: PC_70_BM based X‐ray detectors were fabricated by preparing the P3HT: PC_70_BM solution where 80 mg of P3HT was mixed with 80 mg of PC_70_BM in 1 ml dichlorobenzene (DCB; 1 ml; anhydrous; Sigma–Aldrich). The solution was stirred overnight followed by preheating at 60 °C for 30 min before deposition of the films. The NP‐BHJ X‐ray detectors were fabricated by preparing the P3HT: PC_70_BM: Bi_2_O_3_ solution where 80 mg of P3HT was mixed with 80 mg of PC_70_BM and 80 mg of Bi_2_O_3_ in 1 ml dichlorobenzene (DCB; 1 ml; anhydrous; Sigma‐Aldrich). The solution was stirred overnight followed by preheating at 60 °C for 30 min before deposition of the films. The P3HSe: PC_70_BM based X‐ray detectors were fabricated by preparing the P3HSe: PC_70_BM solution where 80 mg of P3HSe was added to 1 ml of dichlorobenzene heated at 85 °C (DCB; 1 ml; anhydrous; Sigma–Aldrich). The solution was stirred at 85 °C for 3 h to ensure that the P3HSe was completely dissolved. Following this, 80 mg of PC_70_BM was added to the solution. The solution was then stirred overnight at 85 °C before deposition of the films.

### Device Fabrication

Rigid devices were fabricated on ITO (In_2_O_3_: Sn) glass substrates (15 mm × 15 mm, 15 Ω per square, Luminescence Technology Corp.) and curved devices were fabricated on 75 µm thick polyimide substrates (15 mm  ×  15 mm, RS Components) with 120 nm aluminium layer deposited as the cathode using thermal evaporation. An electron transporting Al‐doped ZnO NP dispersion (Sigma–Aldrich) layer was spin coated in the air (3000 rpm for 30 s) and annealed at 80 °C for 10 min to give a thickness of 40 nm. For the P3HT: PC_70_BM based X‐ray detectors, 90 µl of the solution was drop cast to result in a film thickness of 55 µm. For the NP‐BHJ X‐ray detectors, 90 µl of the P3HT: PC_70_BM: Bi_2_O_3_ solution was drop cast to give a film thickness of 55 µm. For the P3HSe: PC_70_BM based X‐ray detectors, 90 µl of the P3HSe: PC_70_BM solution was drop cast to give a film thickness of 55 µm. Devices were annealed (at 60 °C) for ≈60 min in the air, until a relatively dry layer was obtained. After the low temperature annealing process, devices were annealed at 140 °C for 10 min in a N_2_ glove box (MBraun MB20G). This was followed by spin coating of the NiO HTL (2.5 wt.% in Ethanol; Avantama) in the air at 1500 rpm for 30 s to give a film thickness of ≈57 nm. Then the devices were kept under vacuum at a pressure of less than 3 × 10^−3^ mbar for 48 h to remove any residual solvent. Finally, the silver anode (≈120 nm) was deposited by thermal evaporation.

### X‐Ray Irradiation and Characterization

For each detector, three measurements were recorded under each irradiation condition. It should be noted that the dark current response is subtracted from the total photocurrent response during characterization (Equation (2)). The detector response was characterized under soft X‐ray radiation from:

1. A 70 kVp microfocus X‐ray source (Hamamatsu L6732‐01) under a dose rate range of 0.67 – 2.75 cGy s^−1^. A Keithley 2410 source measurement unit was used for recording the electrical characteristics. A dosimeter (Farmer 2670, Thermo Fisher Scientific) in combination with an ionization chamber (2611A, Thermo Fisher Scientific) was used for measurement of dose rates. The dose rates were then calculated for the pixel area of the devices (0.68 cm^2^).
2. An X‐ray therapy system (Gulmay D3225 orthovoltage X‐ray therapy system) which can generate X‐ray energies of 100, 150, and 220 kVp, respectively. A constant dose rate of 68, 70, and 61 cGy min^−1^, respectively were used when irradiated under X‐ray energies of 100, 150, and 220 kVp, respectively. A Keithley 2400 source measurement unit was used for recording the electrical characteristics.
3. A 40 kVp microfocus X‐ray source (Hamamatsu L12161‐07) under a dose rate range of 1.0 – 5.4 mGy s^−1^. A Keithley 2600 source measurement unit was used for recording the electrical characteristics.

### *TRPL*: Glass/ITO/P3HSe

PC_70_BM films and Glass/ITO/NP‐BHJ were prepared as stated earlier. PL decays of the films were measured using a Hamamatsu streak camera in synchroscan mode. The films were excited at ≈20° incidence angle using 200 fs laser pulses at 515 nm and 80 MHz repetition rate with an average laser power density of ≈50 mW cm^−2^. PL was collected from the excitation side to minimize reabsorption. The time resolution of PL decays was ≈2 ps defined as the full width at half maximum of the instrument response function (IRF). Photoluminescence spectra were measured using the same set‐up with the streak camera operating in time‐integrated mode.

### Bendability Characterization

Curved X‐ray detectors were fabricated on polyimide substrates as mentioned earlier. Bendability test on the devices was conducted using an in‐house built uniaxial stretcher in combination with a Python program for position control (actuator speed 1 mm s^−1^). A Keithley 2600 source measurement unit was used for recording the electrical characteristics.

### *Nanoindentation*: Glass/ITO/P3HSe

PC_70_BM films and Glass/ITO/NP‐BHJ were prepared as stated earlier. The Young's modulus and Hardness of each film were measured by an Alemnis Standard Assembly nanoindenter (Alemnis AG). The nanoindentation tests were conducted with a Berkovich (three‐side pyramid) diamond indenter which applied constant loading and unloading rate of 0.2 mN s^−1^ after a load threshold of 0.5 mN. The maximum load was set to 20 mN to ensure a displacement lower than 10% of the film thickness, which was held for 10 s in order to check if the displacement under steady load was lower than ± 10 nm min^−1^. Data analysis was based on the Oliver‐Pharr method.^[^ ^46^ ^]^

### *GIWAXS*: Glass/ITO/P3HSe

PC_70_BM films and Glass/ITO/NP‐BHJ samples were prepared as stated earlier. GIWAXS measurements were performed using a Xeuss 2.0 (Xenocs, France) system equipped with a liquid gallium MetalJet source (Excillum, Sweden) which provides a 9.243 keV (λ = 1.34 Å) X‐ray beam. The beam was collimated to a spot with a lateral dimension of 400 µm on the sample. A Pilatus3R 1 M 2D detector (Dectris, Switzerland) placed at ≈307 mm from the sample was used to obtain the 2D scattering images with both the sample chamber and flight tubes held under vacuum to remove background air scatter. Calibration of the sample‐to‐detector distance was carried out using a silver behenate calibrant in transmission geometry. At incident angles below the critical angle ($\alpha_{c}\approx 0.12^{\circ }$), X‐rays travel as an evanescent plane wave along the sample surface probing the near surface features of the film (the top ≈10 nm) while angles above the critical angle give information about the bulk of the film.^[^ ^36^ ^]^ Therefore, GIWAXS spectra were acquired at incidence angles 0.05° and 0.1° (α_*i*_  <  α_*c*_), as well as 0.2° and 0.3° (α_*i*_ > α_*c*_) to enable probing of the sub‐surface as well as the “bulk” of the blend films (up to ≈2 µm depth). The data were corrected, reduced, and reshaped using the GIXSGUI MATLAB toolbox.^[^ ^47^ ^]^ Parameters for the Scherrer formula were extracted from Gaussian curve fittings of the P3HT (100) peak using OriginPro software. The 1D azimuthally integrated intensity profiles were produced by integrating the 2D GIWAXS patterns across the full *Q* range through various azimuthal (χ) angles normal to the incident beam at the detector; out‐of‐plane (in the *Q_z_* direction −20°< χ <20°), in‐plane (which includes all other angles 20°< χ <90°) and the full χ range.

### Photo‐CELIV, IS, and TPC

The Photo‐CELIV, impedance spectroscopy, intensity modulated photovoltage spectroscopy, intensity dependent impedance spectroscopy, and transient photocurrent measurements were conducted using the Paios 4 all‐in‐one test platform by FLUXiM (Paios 4, Platform for all‐in‐one characterization of solar cells and OLEDs, Fluxim AG 2019, https://www.fluxim.com/paios). Detectors were fabricated using the same method described for X‐ray response characterization. The pixel area was reduced to 3 mm^2^ in order to minimize capacitive effects that can influence device characteristics.

### Cross Sectional Imaging

Samples were prepared by fabrication of P3HSe: PC_70_BM and NP‐BHJ films on the ITO coated glass substrates as described earlier. Cross sectional morphology of each film was examined using a Dual‐beam TESCAN FERA3 microscope; The cross‐sectional milling was carried out using a 30 keV xenon ions (Xe^+^) beam and micrographs were acquired using a mix of secondary and backscattered electrons signal generated after 5 keV electron beam irradiation.

### ToF‐SIMS

Glass/ITO/P3HSe: PC_70_BM films and Glass/ITO/NP‐BHJ samples were prepared as stated earlier. Time‐of‐flight secondary ion mass spectrometry was used for depth profiling of the P3HSe: PC_70_BM and NP‐BHJ film using a TOF.SIMS 5 (IONTOF GmbH) instrument with 30 keV $Bi_{3}^{+}$ ions for analysis and $Ar_{1700-2000}^{+}$ ions at 20 keV for sputtering in non‐interlaced mode. Low‐energy electrons were used to flood the surface during the measurements. The sputter beam was rasterized in random mode over a 250 × 250 µm^2^ area with the analysis area of 50 × 50 µm^2^. Every plane of the analyzed volume was rasterized in random mode with 128 × 128 pixels at 1 shot per pixel and 3 frames followed by 7 frames of sputtering. The instrument was operated in the negative ion polarity.

Data acquisition was obtained using SurfaceLab software (IONTOF GmbH). Depth profiles were exported using the ASCII export function and 3D maps were exported using the 3D Rendering function.

### Energy Deposition Simulations

FLUKA, a Monte Carlo simulation program designed for the interaction and transport of particles and nuclei in matter, was used for simulation of the X‐rays interaction with the active material. To simulate the energy depletion of the devices, the geometry of the P3HSe: PC_70_BM and NP‐BHJ films were modelled and irradiated with 38 keV X‐ray photons to be processed by the FLUKA program.

### TEM Analysis

Transmission electron microscopy (TEM) analysis was carried out in a field emission Talos F200i, Thermo Scientific, using a 200 keV beam. The sample was prepared by sonicating the powder for 20 min in an isopropyl alcohol (IPA) solution. A 10 µl volume of the dispersion was drop cast in a holey carbon support film and UV‐ozone cleaned for 3 min. Images analyses were done with ImageJ software where an intermodes color threshold method was used to calculate particles area and fast‐Fourier transform allowed to measure interplanar spacings. The particle frequency distribution was processed using the descriptive statistics tool in Origin2021 software.

## Conflict of Interest

K.D.G.I.J., and S.R.P.S. are inventors on a patent (Direct Conversion Radiation Detector, International Publication Number: WO2018/078372A1) which is assigned to SilverRay Ltd.

## Supporting information

Supporting Information

## Data Availability

The data that support the findings of this study are available from the corresponding author upon reasonable request.
