# Supplementary material for: Tissue Equivalent Curved Organic X‐ray Detectors Utilizing High Atomic Number Polythiophene Analogues
Source: Adv Sci (Weinh). 2023 Nov 2;10(35):2304261. doi: 10.1002/advs.202304261 (PMC10724441; doi:10.1002/advs.202304261)
Supplement: Supplementary file 1 — Supporting Information [file ADVS-10-2304261-s001.pdf]

ADVANCED SCIENCE
Open Access

Supporting Information

for *Adv. Sci.*, DOI 10.1002/advs.202304261

Tissue Equivalent Curved Organic X-ray Detectors Utilizing High Atomic Number Polythiophene Analogues

*M. Prabodhi A. Nanayakkara, Qiao He, Arvydas Ruseckas, Anushanth Karalasingam, Lidija Matjacic, Mateus G. Masteghin, Laura Basiricò, Ilaria Fratelli, Andrea Ciavatti, Rachel C. Kilbride, Sandra Jenatsch, Andrew J. Parnell, Beatrice Fraboni, Andrew Nisbet, Martin Heeney, K. D. G. Imalka Jayawardena and S. Ravi P. Silva**

© Copyright 2023. WILEY-VCH GmbH.

Supporting Information

Tissue equivalent curved organic X-ray detectors utilising high atomic number polythiophene analogues

*M. Prabodhi A. Nanayakkara, Qiao He, Arvydas Ruseckas, Anushanth Karalasingam, Lidija Matjacic, Mateus G. Masteghin, Laura Basiricò, Ilaria Fratelli, Andrea Ciavatti, Rachel C. Kilbride, Sandra Jenatsch, Andrew J. Parnell, Beatrice Fraboni, Andrew Nisbet, Martin Heeney, K. D. G. Imalka Jayawardena, S. Ravi P. Silva**

Dr M. P. A. Nanayakkara, Dr M. G. Masteghin, Dr K. D. G. I. Jayawardena, Prof. S. R. P. Silva
Advanced Technology Institute, Department of Electrical and Electronic Engineering, University of Surrey, Guildford, Surrey, GU2 7XH, United Kingdom.
E-mail: s.silva@surrey.ac.uk

Prof. M. Heeney, Dr Q. He
Department of Chemistry and Centre for Processable Electronics, Imperial College London, White City Campus, London W12 0BZ, United Kingdom.

Dr. A. Ruseckas
School of Physics & Astronomy, University of St Andrews, Physical Science Building, North Haugh, St Andrews, United Kingdom.

Mr. A. Karalasingam
Sri Lanka Institute of Nanotechnology, Pitipana - Thalagala Rd, Homagama, Sri Lanka.

Dr L. Basiricò, Dr I. Fratelli, Dr A. Ciavatti, Prof. B. Fraboni
Department of Physics and Astronomy, University of Bologna, Viale Berti Pichat 6/2, Bologna 40127, Italy.
National Institute for Nuclear Physics, INFN section of Bologna, Bologna, Italy.

Dr R. C. Kilbride
Department of Chemistry, University of Sheffield, Dainton Building, Sheffield, S3 7HF, United Kingdom

Dr A. J. Parnell
Department of Physics and Astronomy, University of Sheffield, Hicks Building, Sheffield, S3 7RH, United Kingdom.

Dr S. Jenatsch
FLUXiM AG, Katharina-Sulzer-Platz 2, 8400 Winterthur, Switzerland.

Dr. L. Matjacic
National Physical Laboratory, Teddington, Middlesex, TW11 0LW, United Kingdom.

Prof. A. Nisbet
Department of Medical Physics and Biomedical Engineering, University College London, Gower St, Bloomsbury, London WC1E 6BT, UK.

Note S1. Estimation of the $W_{\pm}$ for a semiconductor using the Klein rule

X-ray detectors are expected to possess as high sensitivity as possible. Therefore, it must be able to produce as many as free EHP as possible per unit of absorbed X-ray radiation. This is influenced by $W_{\pm}$ which should be as low as possible since the total collectable charge (ΔQ) generated from the absorbed X-ray radiation is:

$$\Delta Q = e\Delta E/W_{\pm} \tag{S1}$$

Where e is the elementary charge.

For semiconductors, the $W_{\pm}$ required to create an EHP has been shown to depend on the energy bandgap E_g via Klein rule[26]:

$$W_{\pm} \approx 2.8E_g + E_{phonon} \tag{S2}$$

where E_{phonon} is the energy of a phonon which is taken roughly as ~0.5 eV.[26]

Note S2. Influence of the distance on the charge carrier recombination probability

In the absence of external fields, the probability that the charge pair escapes recombination is given by a well-known formula by Onsager[48]:

$$\emptyset_0(r_0) = exp\left(-\frac{r_c}{r_0}\right) \tag{S3}$$

Where r_0 is the initial distance between the charges and r_c is the Onsager radius defined by $r_c = e^2/\varepsilon k_B T$. Here, e denotes the electron charge, ε is the dielectric constant, k_B is the Boltzmann constant, and T is the temperature.

In the presence of an external electric field, the charge pairs in an ionized system are additionally separated by the field, and the escape probability further increases.

Note S3. Estimation of the average P3HT and P3HSe CCL using the Scherrer formula

The average P3HT and P3HSe CCL can be estimated (Table S2, Supporting Information) using the Scherrer formula[36] (equation S2) on the P3HT (100) and P3HSe (100) out-of-plane lamellar peak observed in the 1D azimuthally integrated intensity profiles (Figure S5 and 3b):

$$D = \frac{K\lambda}{\beta\cos(\theta)} \tag{S4}$$

where D is the CCL, K is the dimensionless shape factor (0.94), λ is the X-ray wavelength (0.134 nm), β is the full width at half maximum of the P3HT (100) and P3HSe (100) peak in radians, and θ is the Bragg angle in radians.

Note S4. Estimation of the Young's modulus of the blend films

The Young's modulus, E of both films can be estimated based on the model proposed by Oliver and Pharr:

$$E = \frac{(1-\upsilon^2)}{\frac{1}{E_r} - \frac{(1-\upsilon_i^2)}{E_i}} \tag{S5}$$

where E_r is the reduced modulus given by $S\sqrt{pi}/2\sqrt{\grave{A}}$, and S represents the contact stiffness (dP/dh) estimated from the slope of the initial unloading curve, and $\grave{A}$ represents the real projected contact area of the indenter which is defined by the Berkovich tip and the contact depth (h_c). υ and v_i are the Poisson's ratio of the sample and the indenter (diamond). For polymeric films, the Poisson's ratio is taken as 0.3[49] and the same value was used for both P3HSe: $PC_{70}BM$ film and NP-BHJ film during this analysis. The Poisson's ratio for the indenter is equal to 0.07.[50] The E_i in equation (8) which represents the Young's modulus of the indenter was reported as 1141 GPa.[50]

Note S5. Estimation of the hardness of the blend films

The hardness (H) of each film can be estimated using:

$$H = \frac{P_{max}}{\hat{A}} \quad \text{(S6)}$$

where P_{max} is the maximum value of applied load (10 mN) and $\hat{A}$ is the real projected contact area of the indenter which is given as $24.56 h_c^2$.[49]

Note S6. Effect of Bending Radius on Beam Area Interacting with the Detector

As reported in our previous study,[6] it should be noted that when a planar detector is used, the incident beam is interacting with the entire pixel area of the detector. However, when the detector is bent to a certain radius of curvature (R), the beam area interacting with the detector is reduced. For pixel with a length L (= 8 mm for this study), width w (= 8 mm for this study), and the angle (θ) between the beam axis and the pixel width (Figure X), the resulting reduced beam area is ($\overline{\mathrm{A}}$) is given by:

$$\overline{\mathrm{A}} = 2\mathrm{R} \sin\theta \times \mathrm{L} \quad \text{(S7)}$$

where, θ is given by $^{\mathrm{w}}/_{2\mathrm{R}}$. This results in a lower dose/dose rate being incident upon the detector when smaller curvature radius is reached.

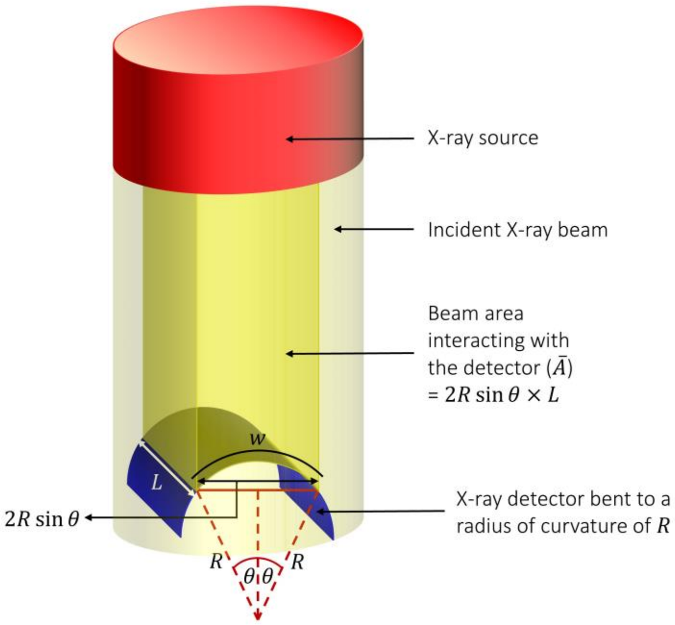

Figure S21. Schematic diagram illustrating the variation of the beam area interacting with the detector with the bending radius of curvature.

Table S1. Materials and dimensions of each layer for the simulated detectors

P3HSe: $PC_{70}BM$ X-ray detector					NP-BHJ X-ray detector				
Identifier	Material	Dimension (cm)			Identifier	Material	Dimension (cm)		
		x	y	z			x	y	z
1	Glass	0.4	0.425	0.5	1	Glass	0.4	0.425	0.5
2	ITO	0.4	0.425	2×10^{-5}	2	ITO	0.4	0.425	2×10^{-5}
3	Al-doped ZnO	0.4	0.425	4×10^{-6}	3	Al-doped ZnO	0.4	0.425	4×10^{-6}
4	P3HSe: $PC_{70}BM$	0.4	0.425	5.5×10^{-3}	4	P3HT: $PC_{70}BM$: Bi_2O_3	0.4	0.425	2.75×10^{-3}
5	NiO	0.4	0.425	5.7×10^{-6}	5	P3HT: $PC_{70}BM$	0.4	0.425	2.75×10^{-3}
6	Ag	0.4	0.425	1.2×10^{-5}	6	NiO	0.4	0.425	5.7×10^{-6}
			0.425		7	Ag	0.4	0.425	1.2×10^{-5}

Table S2. Variation of d-spacing, FWHM, and CCL with the incident angle for the P3HT: $PC_{70}BM$, P3HT: $PC_{70}BM$: Bi_2O_3, and P3HSe: $PC_{70}BM$ blend films. Parameters have been estimated using GIWAXS data.

Blend film	Angle (°)	d-spacing (2π/Q) (nm)	FWHM β (degrees)	Crystalline coherence length D (nm)
P3HT: $PC_{70}BM$	0.05	1.51	0.39	18.6
	0.1	1.51	0.416	17.4
	0.2	1.52	0.407	17.7
	0.3	1.52	0.404	17.9
P3HT: $PC_{70}BM$: Bi_2O_3	0.05	1.52	0.44	16.4
	0.1	1.52	0.432	16.7
	0.2	1.52	0.42	17.2
	0.3	1.52	0.4	18.1
P3HSe: $PC_{70}BM$	0.05	1.43	0.523	13.8
	0.1	1.44	0.531	13.6
	0.2	1.44	0.537	13.5
	0.3	1.44	0.508	14.2

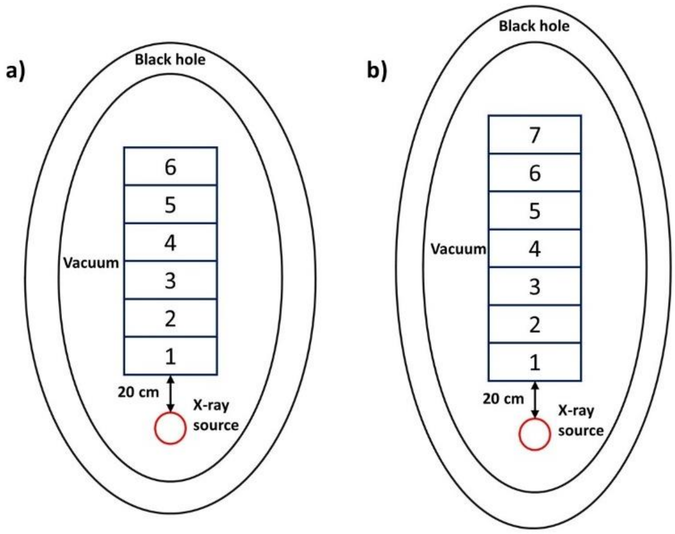

Figure S1. Schematic of the geometrical model used for FLUKA energy deposition simulations of the a) P3HSe: $PC_{70}BM$ based detector, b) NP-BHJ X-ray detector.

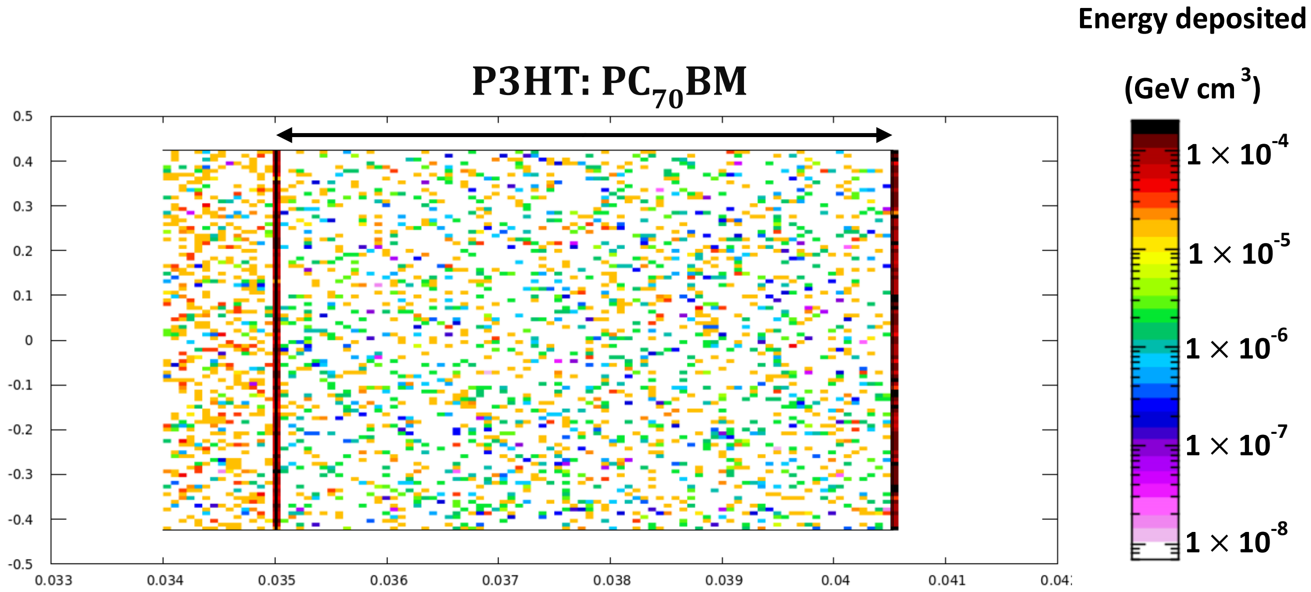

Figure S2. Energy deposited in the P3HT: $PC_{70}BM$ blend film simulated using the FLUKA software (http://www.fluka.org/fluka.php).

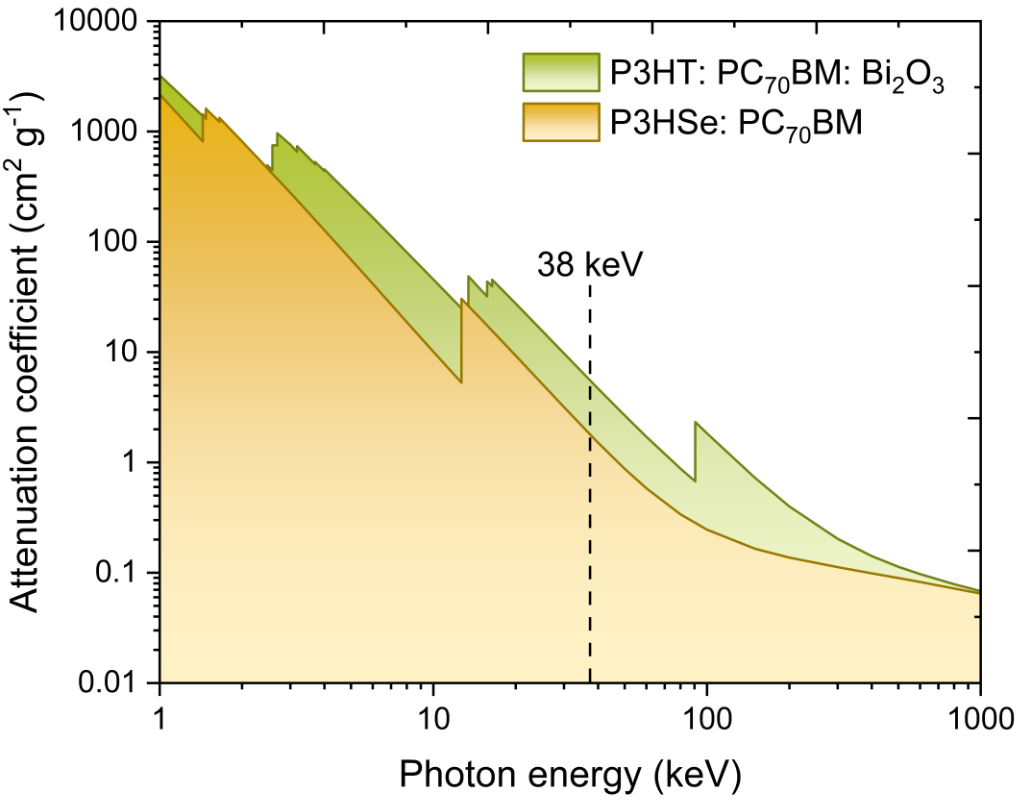

Figure S3. The mass attenuation coefficient for the P3HT: $PC_{70}BM$: Bi_2O_3 and P3HSe: $PC_{70}BM$ blend films generating using NIST XCOM (https://physics.nist.gov/PhysRefData/Xcom/html/xcom1.html). The dashed line indicates the 38 keV X-ray energy.

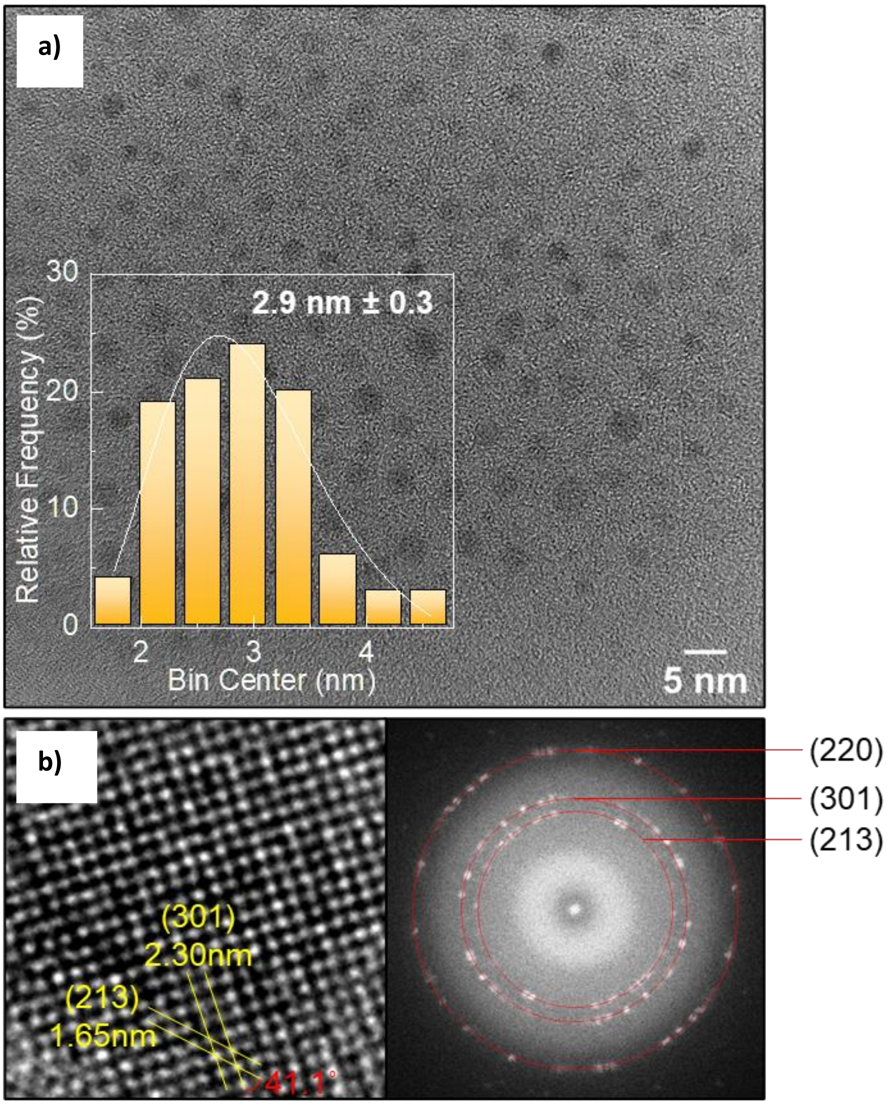

Figure S4. Particle size of Bi_2O_3 nanoparticles. (a) a low-magnification TEM image of the Bi_2O_3 particles dispersed on the top of the carbon film. The relative frequency distribution of particles diameter shows an average size of about 3nm, although some regions of the grid present considerable agglomeration similar to that observed in the NP-BHJ film (Figure 1a). (b) A high-resolution transmission electron microscopy (HR-TEM) image of the Bi_2O_3 particles. This enabled to measure interplanar spacings of 2.30 nm and 1.65 nm that based on the JCPDS 27-50 of tetragonal β -Bi_2O_3 phase has been indexed as planes (301) and (213), respectively, both of which form an angle of 41.1° between them. Selected area electron diffraction (SAED) further confirms the presence of a β -Bi_2O_3 phase.

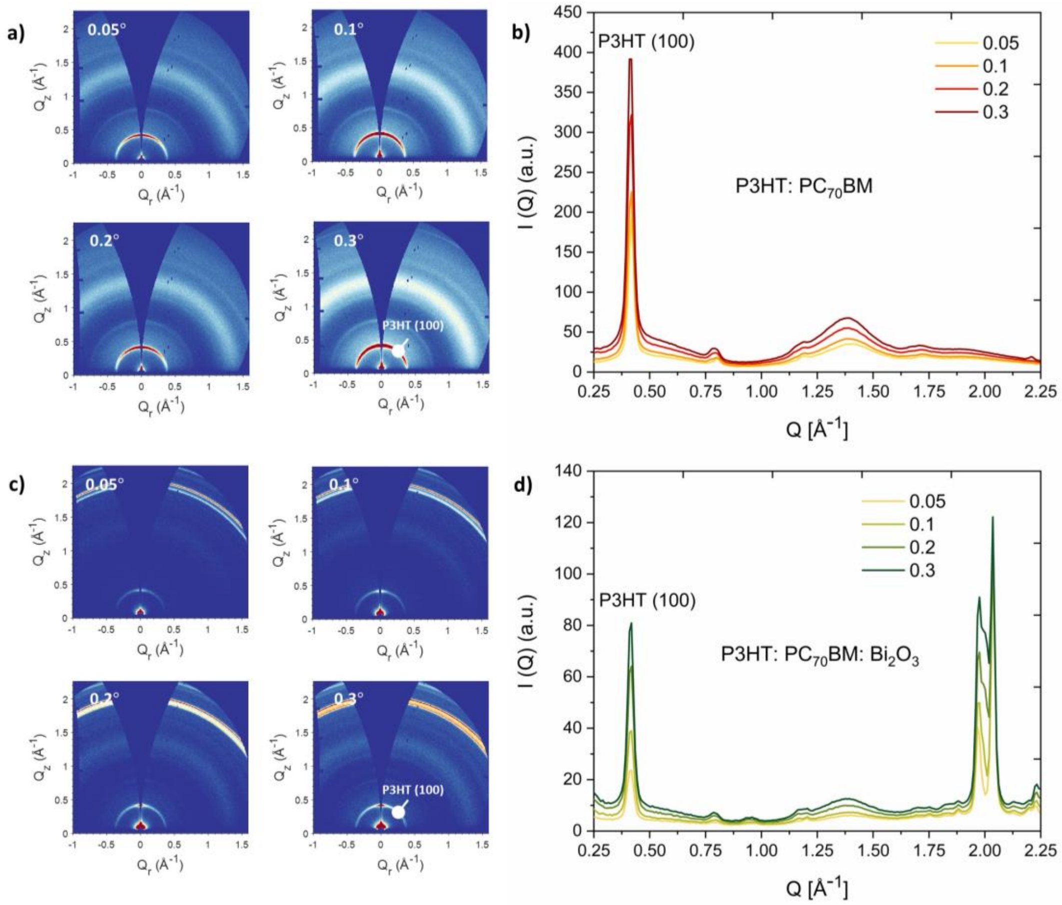

Figure S5. 2D GIWAXS spectra obtained at 0.05°, 0.1°, 0.2°, and 0.3° incident angles for the (a) P3HT: $PC_{70}BM$ and (c) P3HT: $PC_{70}BM$: Bi_2O_3 blend films. 1D azimuthally integrated intensity profiles of the (b) P3HT: $PC_{70}BM$ and (d) P3HT: $PC_{70}BM$: Bi_2O_3 blend film across the full azimuthal range for various incidence angles.

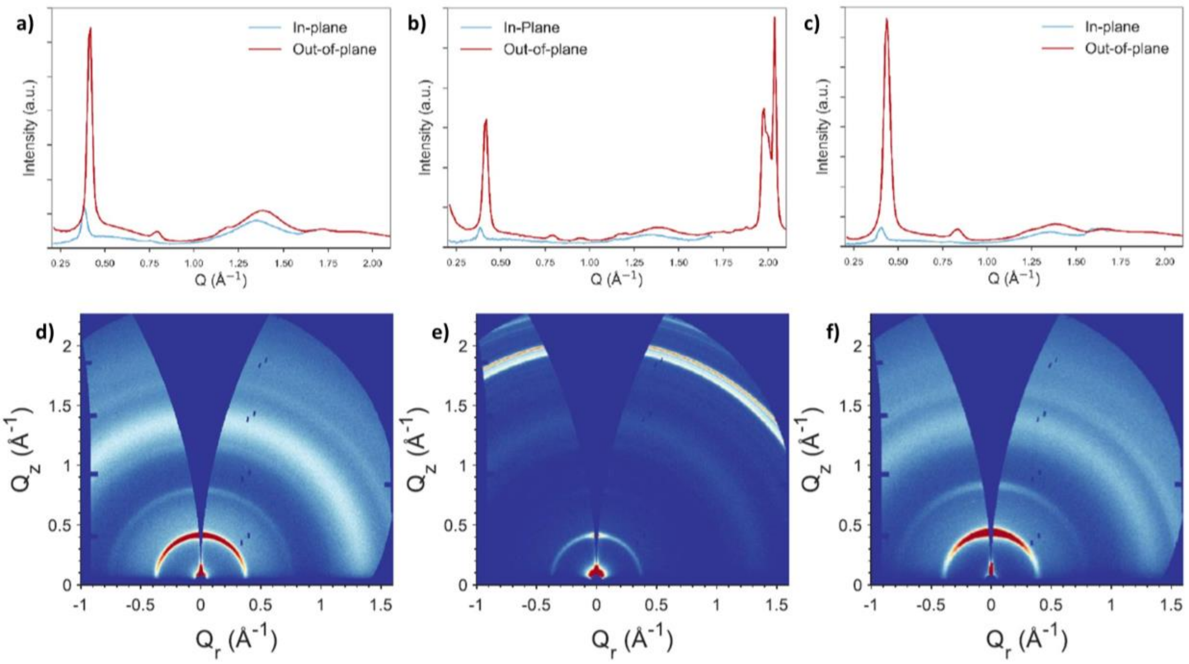

Figure S6. Comparison of in-plane and out-of-plane 1D intensity profiles of the (a) P3HT: $PC_{70}BM$, (b) P3HT: $PC_{70}BM$: Bi_2O_3, and (c) P3HSe: $PC_{70}BM$ blend films at an incidence angle of 0.3°. Comparison of the 2D GIWAXS spectra of the (d) P3HT: $PC_{70}BM$, (e) P3HT: $PC_{70}BM$: Bi_2O_3, and (f) P3HSe: $PC_{70}BM$ blend films at an incidence angle of 0.3°.

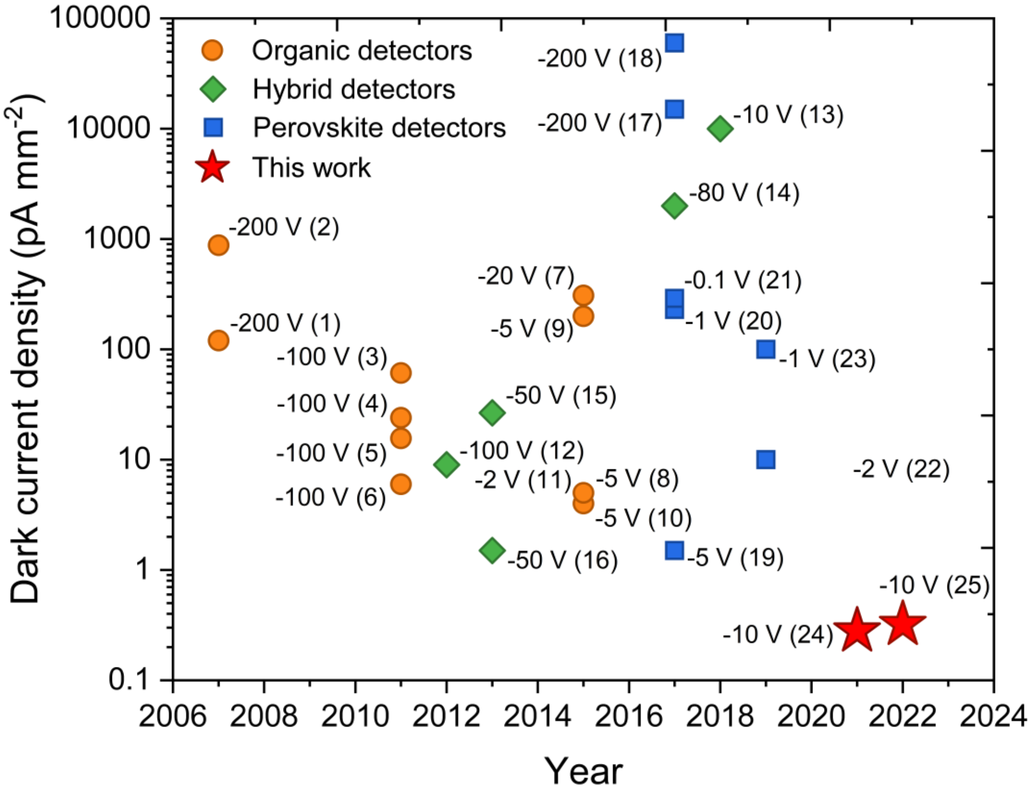

Figure S7. Comparison of the dark current response of the organic detectors ((1)-(2),[51] (3)-(6),[10] (7),[9] (8)-(10),[52] and (11)[53]), high-Z NP sensitized hybrid detectors ((12),[54] (13),[11] (14),[55] (15)-(16)[56]), perovskite detectors ((17),[57] (18),[58] (19),[59] (20)-(221,[60] (22),[61] and (23)[62]), NP-BHJ detector (24) and the P3HSe: $PC_{70}BM$ detector fabricated in this work (25).

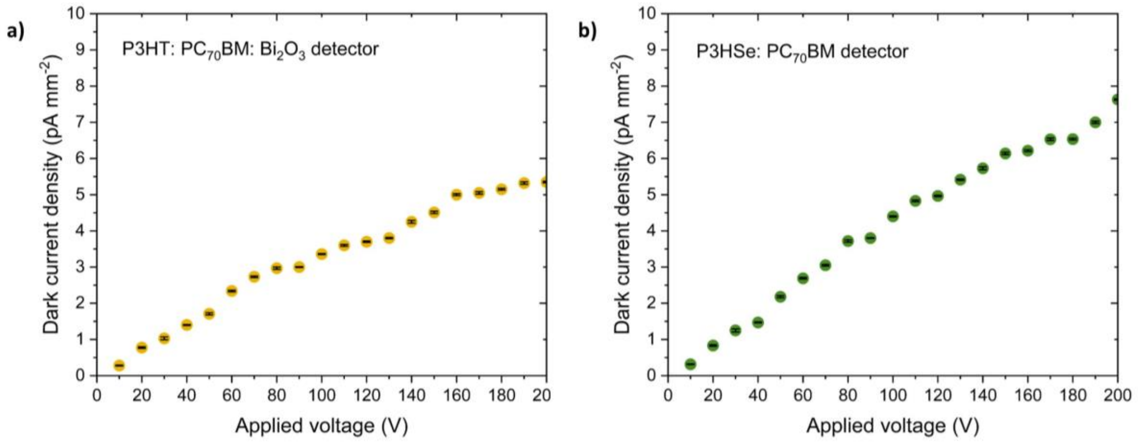

Figure S8. The dark current density as a function of applied bias for the a) NP-BHJ and b) P3HSe: $PC_{70}BM$ X-ray detector. The data points in the figure are averaged over three separate detector measurements.

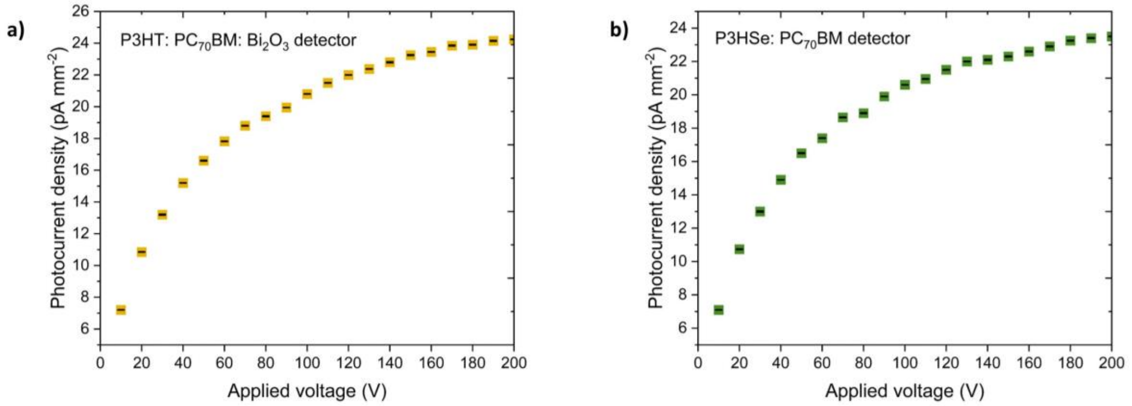

Figure S9. The photocurrent density as a function of applied bias for the a) NP-BHJ and b) P3HSe: $PC_{70}BM$ X-ray detector. The data points in the figure are averaged over three separate detector measurements.

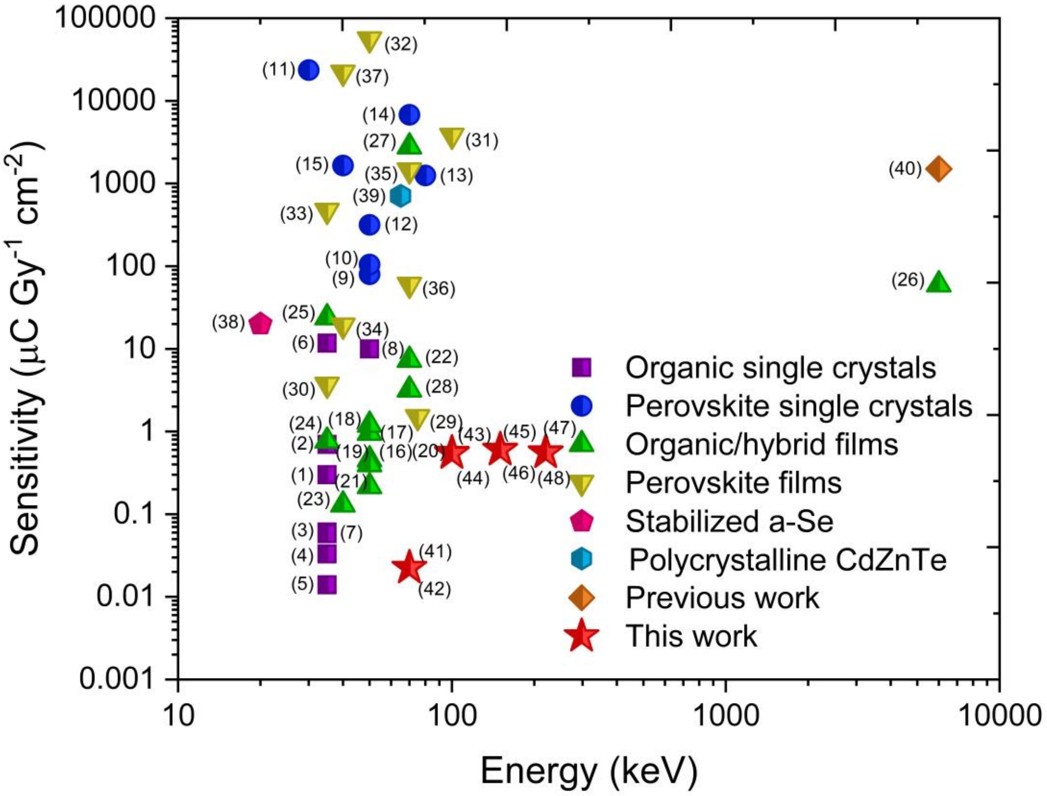

Figure S10. Comparison of the sensitivity of the organic single crystals ((1),[8] (2)-(3),[63] (4)-(5),[52] (6),[9] (7),[64] (8)[65]), perovskite single crystals ((9),[66] (10),[59] (11),[67] (12),[68] (13),[69] (14),[70] and (15)[71]), organic/hybrid films ((16)-(17),[51] (18),[7] (19),[10] (20),[54] (21),[56] (22),[14] (23),[72] (24),[39] (25),[55] (26),[11] (27),[40] and (28)[73]), perovskite films ((29),[74] (30),[75] (31),[57] (32),[76] (33),[61] (34),[77] (35),[78] (36),[79] and (37)[80]), stabilized amorphous Selenium (a-Se) detectors (38),[43] polycrystalline cadmium zinc telluride (CdZnTe) detectors (39),[81] ultra-low dark current detectors introduced in our previous work (40)[12], and the NP-BHJ ((41), (43), (45), and (47)) and P3HSe: $PC_{70}BM$ detectors fabricated in this work ((42), (44), (46), and (48)).

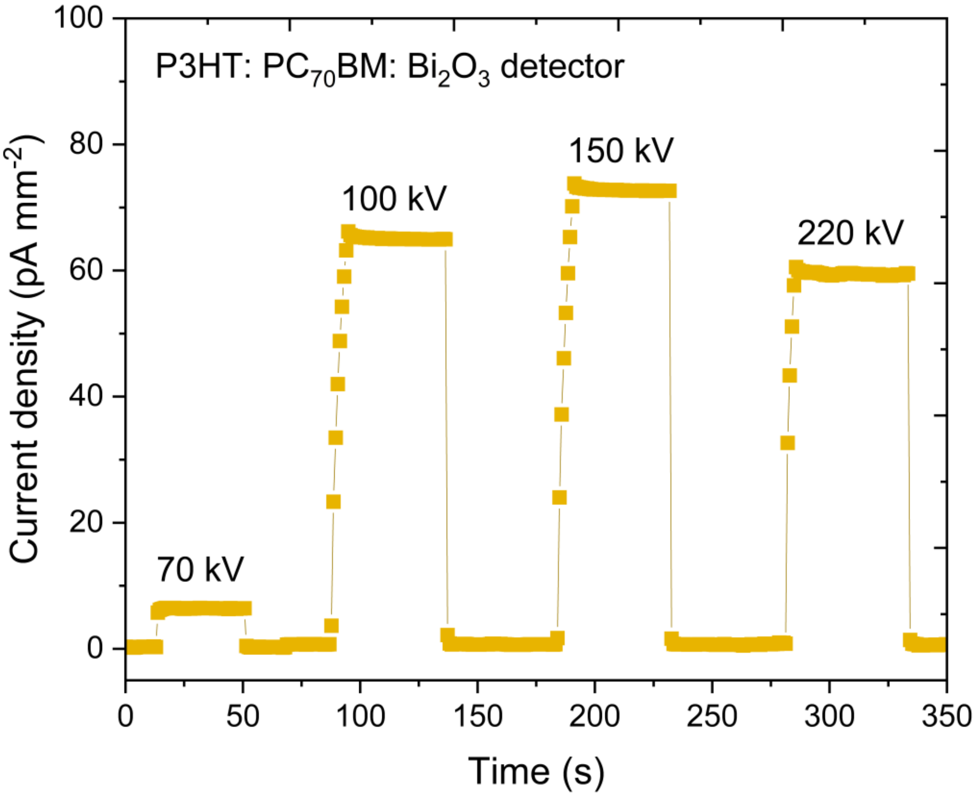

Figure S11. Transient X-ray photocurrent response from the P3HT: $PC_{70}BM$: Bi_2O_3 detector under the different X-ray energies indicating a "square shaped" response.

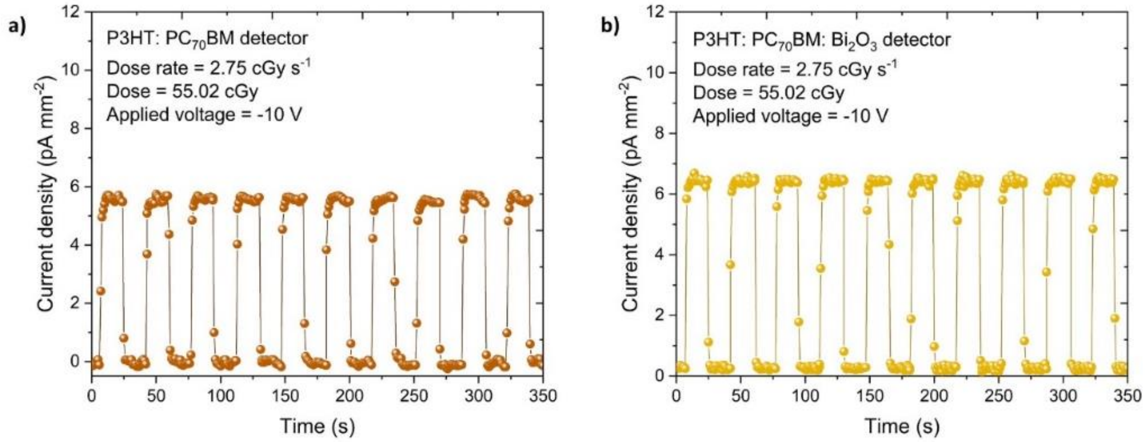

Figure S12. Reproducibility of the photocurrent response of the X-ray detectors based on a) P3HT: $PC_{70}BM$, b) P3HT: $PC_{70}BM$: Bi_2O_3 blend films under the 70 kVp X-ray radiation.

Figure S13. (a) Overtime stability of the dark current and photocurrent response of the detector. (b) Photocurrent response from the detector under a series of X-ray exposures

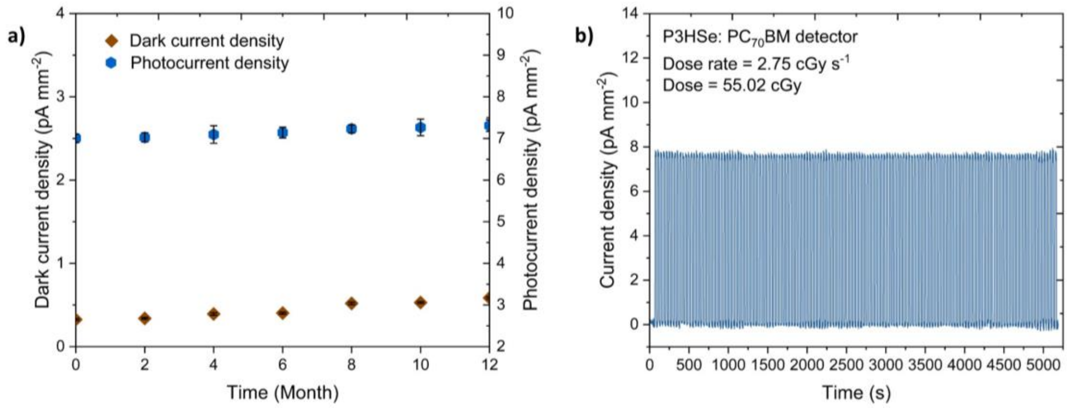

resulting in a cumulative dose of 100 Gy. The detector response doesn't display any significant degradation indicating the excellent radiation stability of the P3HSe: $PC_{70}BM$ system. The data points in figure (a) are averaged over three separate detector measurements.

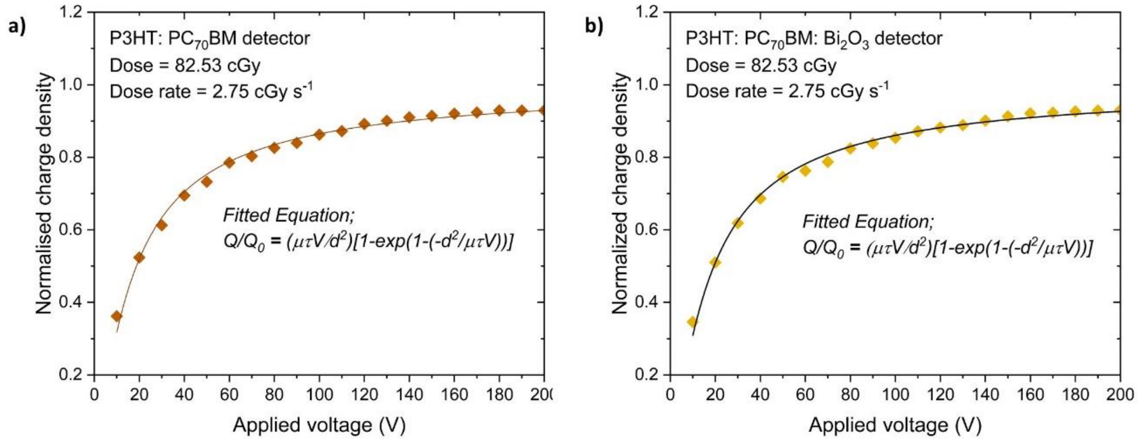

Figure S14. Voltage dependence together with the Hecht fit ($R^2 > 0.9998$) of the X-ray detectors based on a) P3HT: $PC_{70}BM$, b) P3HT: $PC_{70}BM$: Bi_2O_3 blend films.

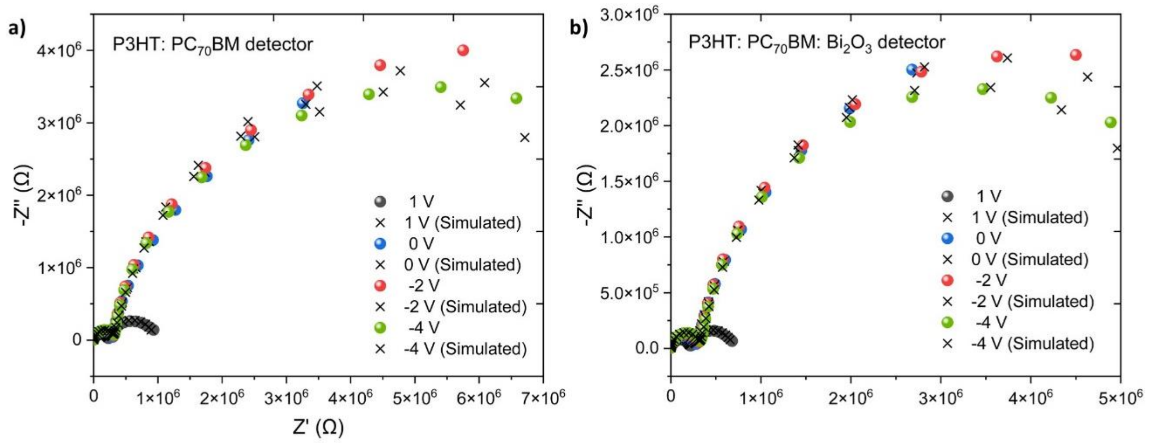

Figure S15. Nyquist plots of the a) P3HT: $PC_{70}BM$, b) P3HT: $PC_{70}BM$: Bi_2O_3 based detector under dark conditions when biased at +1, 0, -2, -4 V. The black colour crosses (×) represent

the fits for each bias calculated using the equivalent circuit shown in the inset. R_1 and R_2 are resistance components forming a parallel circuit with the constant phase elements CPE_1 and CPE_2.

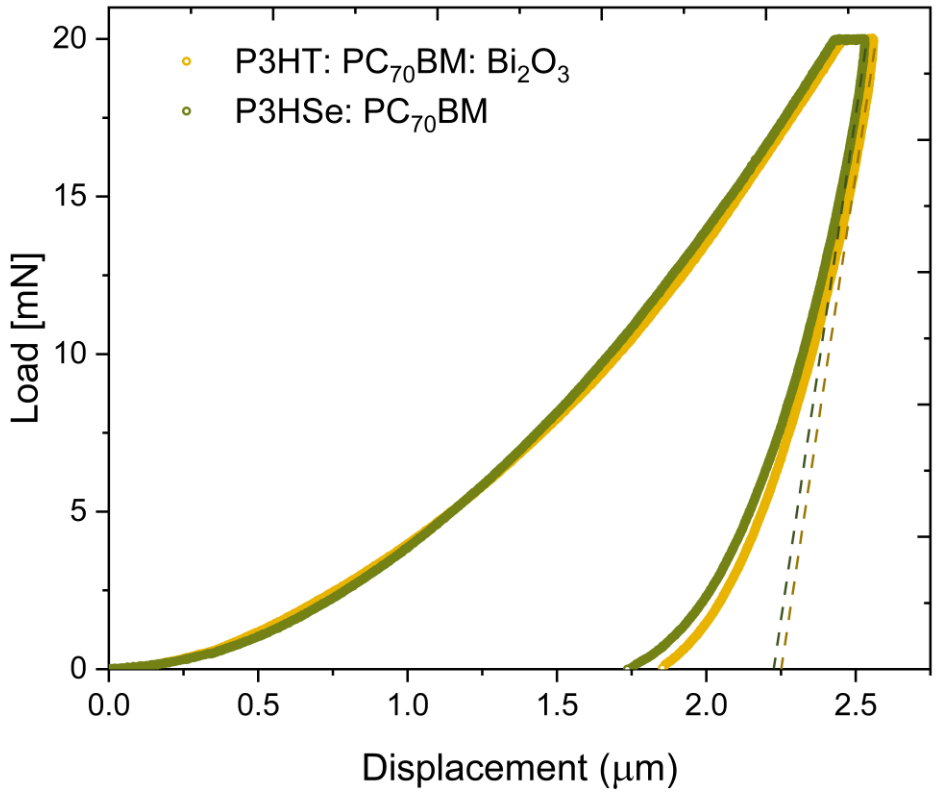

Figure S16. Loading and unloading P–h curves for the P3HSe: $PC_{70}BM$ and NP-BHJ film.

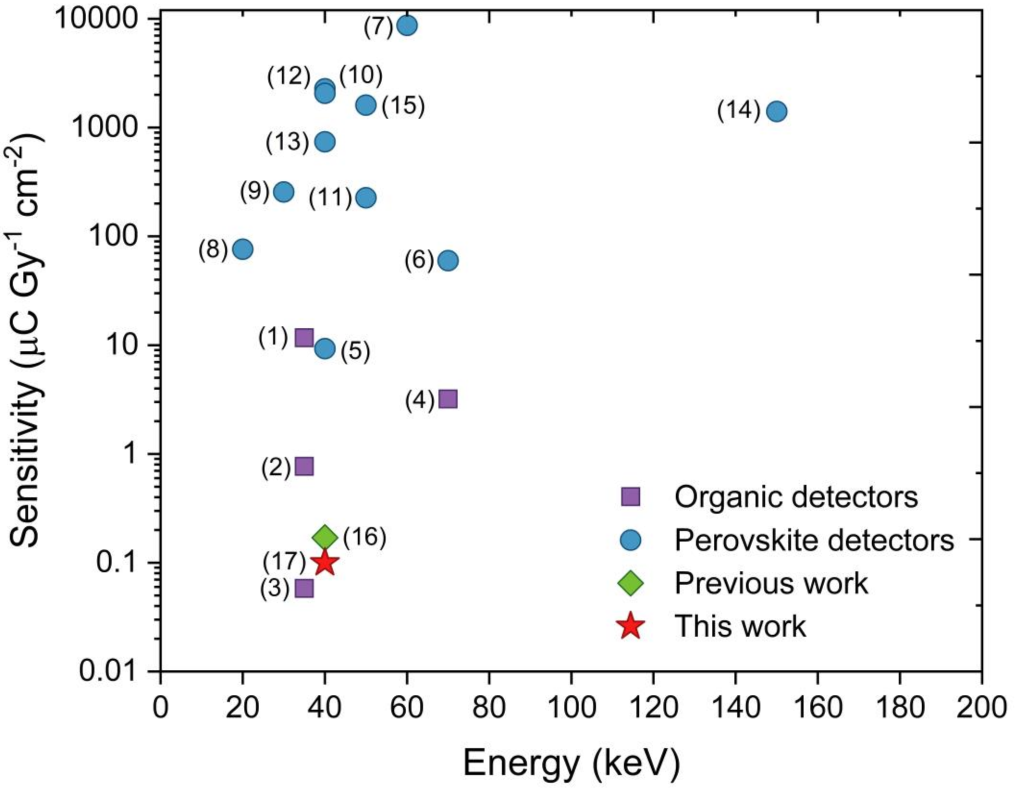

Figure S17. Comparison of the sensitivity of the organic detectors ((1),[9] (2),[39] (3),[64] and (4)[73]), perovskite detectors ((5),[82] (6),[79] (7),[83] (8),[84] (9),[15] (10),[85] (11),[15] (12),[86] (13),[15] (14),[87] and (15)[88]), curved NP-BHJ detectors introduced in our previous work (16)[6], and P3HSe: $PC_{70}BM$ detectors fabricated in this work (17).

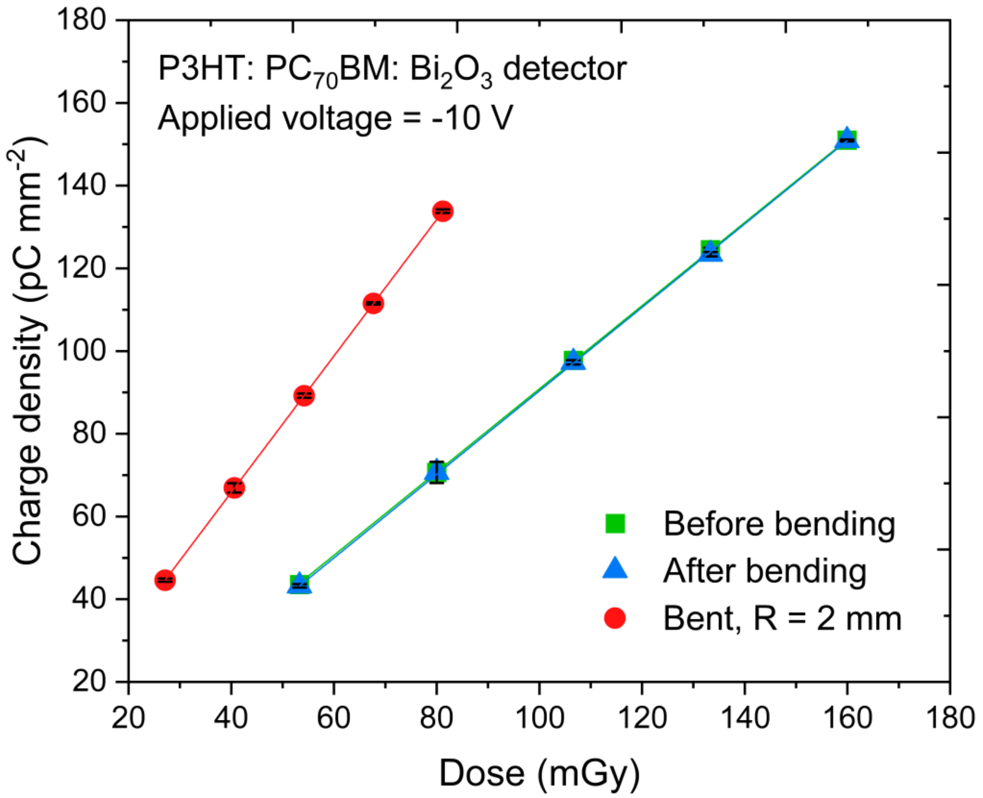

Figure S18. Charge density as a function of the incident dose for the P3HT: $PC_{70}BM$: Bi_2O_3 detector measured before bending (green solid squares), during bending with a bending radius of 2 mm (red solid circles), and after bending (blue solid triangles) indicating dose linearity under each condition. The data points in the figure are averaged over three separate detector measurements.

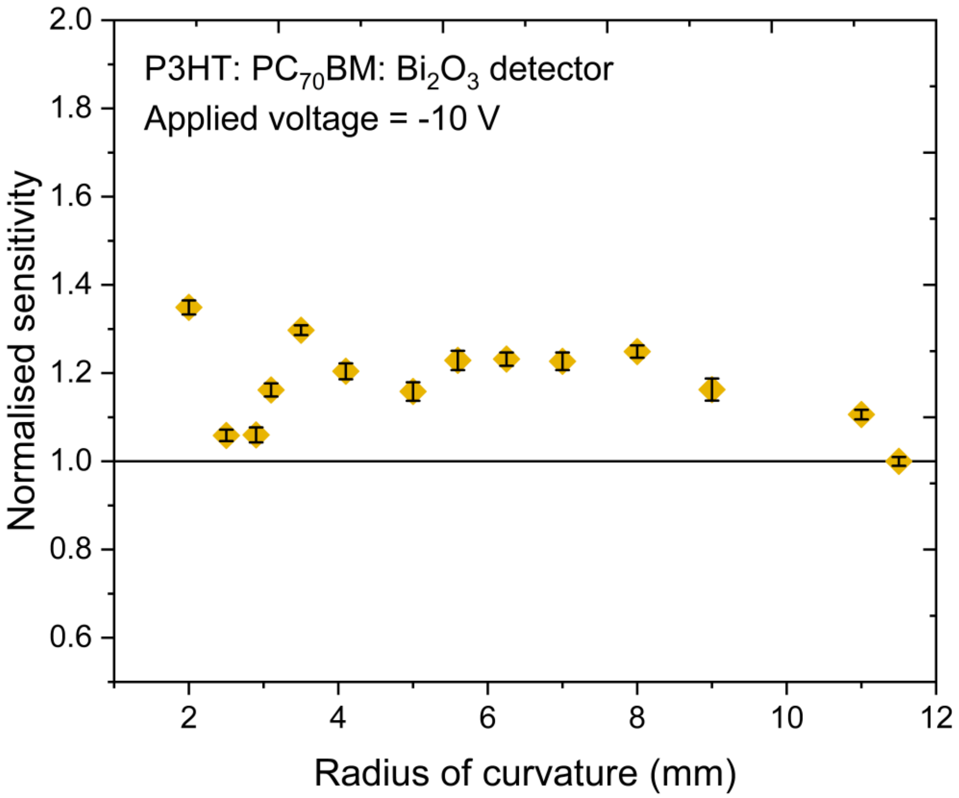

Figure S19. Normalised sensitivity of the P3HT: $PC_{70}BM$: Bi_2O_3 detector as a function of bending radius indicating the threshold curvature radius limit for bendability. The data points in the figure are averaged over three separate detector measurements.

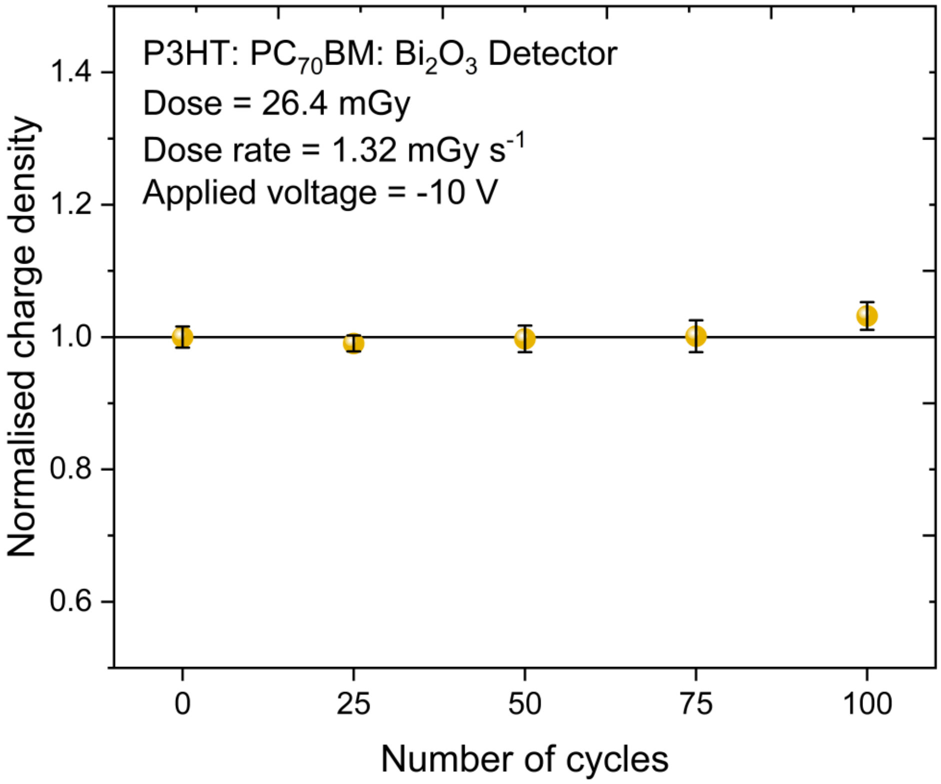

Figure S20. Normalised charge density of the P3HT: $PC_{70}BM$: Bi_2O_3 detector measured before bending, and after 25, 50, 75, and 100 bending cycles. The data points in the figure are averaged over three separate detector measurements.
